# Characterization of the heterogeneity in SARS-CoV-2 fitness dynamics via graph representation learning

**DOI:** 10.1371/journal.pcbi.1013582

**Published:** 2026-01-12

**Authors:** Zengmiao Wang, Ziqin Zhou, Junfu Wang, Lingyue Yang, Zhirui Zhang, Weina Xu, Zeming Liu, Yuxi Ge, Liang Yang, Xiaoli Wang, Peng Yang, Quanyi Wang, Yunlong Cao, Yuanfang Guo, Huaiyu Tian

**Affiliations:** 1 Beijing Key Laboratory of Surveillance, Early Warning and Pathogen Research on Emerging Infectious Diseases, Beijing Research Center for Respiratory Infectious Diseases, State Key Laboratory of Remote Sensing and Digital Earth, Center for Global Change and Public Health, Beijing Normal University, Beijing, China; 2 School of Computer Science and Engineering, Beihang University, Beijing, China; 3 School of Statistics, Beijing Normal University, Beijing, China; 4 School of Artificial Intelligence, Hebei University of Technology, Tianjin, China; 5 Beijing Key Laboratory of Surveillance, Early Warning and Pathogen Research on Emerging Infectious Diseases, Beijing Center for Disease Prevention and Control, Beijing, China; 6 Beijing Research Center for Respiratory Infectious Diseases, Beijing, China; 7 School of Public Health, Capital Medical University, Beijing, China; 8 Biomedical Pioneering Innovation Center, Peking University, Beijing, China; 9 School of Life Sciences, Peking University, Beijing, China; 10 Peking-Tsinghua Center for Life Sciences, Tsinghua University, Beijing, China; 11 National Key Laboratory of Intelligent Tracking and Forecasting for Infectious Diseases, Joint International Research Laboratory of Catastrophe Simulation and Systemic Risk Governance, School of National Safety and Emergency Management, Beijing Normal University, Beijing, China; Beijing Institute of Genomics Chinese Academy of Sciences, CHINA

## Abstract

Understanding the heterogeneity of population-level viral fitness dynamics, which reflect the interplay between intrinsic viral properties and population immunity, is critical for pandemic preparedness. However, how these dynamics vary across diverse immune backgrounds and mutational landscapes remain poorly characterized. We present Geno-GNN, a graph representation learning approach for retrospectively characterizing the viral fitness dynamics of severe acute respiratory syndrome coronavirus 2 (SARS-CoV-2). Geno-GNN accurately predicts angiotensin-converting enzyme 2 (ACE2) binding affinity and immune escape potential across multiple external datasets. Using Geno-GNN, we identified temporal patterns in SARS-CoV-2 fitness and detected varying rates of fitness change associated with distinct immune backgrounds. Virtual mutation scanning revealed two fitness trajectories: broad immune evasion at the cost of ACE2 affinity and ACE2 affinity maintenance at or above the Wuhan-Hu-1 level along with moderate immune escape. Notably, real-world SARS-CoV-2 variants predominantly followed the latter trajectory, sustaining ACE2 affinity via fixed mutations. These findings underscore the heterogeneous, immune-contextualized nature of viral fitness dynamics and the complex evolutionary pathways of SARS-CoV-2.

## Author summary

Understanding how viruses adapt to population immunity is essential for managing pandemics. Here, we developed Geno-GNN, a deep learning framework that accurately predicts two critical viral fitness from sequence data: the ability to bind to human cells and the capacity to evade immunity. Applying Geno-GNN to SARS-CoV-2, we found that the rate of change in its fitness varied in response to different contexts of population immunity. Two fitness strategies were identified: sacrificing infectivity for stronger immune escape, or maintaining infectivity while achieving moderate immune evasion. Real-world SARS-CoV-2 variants have followed the latter, balanced trajectory, maintained by the synergistic effect of multiple mutations acting in concert. By capturing these complex interaction, Geno-GNN provides a useful tool to systematically explore viral sequence space and to support preparedness for future emerging pathogens.

## Introduction

The coronavirus disease 2019 (COVID-19) pandemic has been characterized by the rapid evolution of severe acute respiratory syndrome coronavirus 2 (SARS-CoV-2), driven by the accumulation of adaptive mutations [1,2]. These mutations have defined distinct viral variants [3–5], each exhibiting unique fitness characteristics. For example, the Alpha (B.1.1.7) and Beta (B.1.351) variants demonstrate increased affinities for the human angiotensin-converting enzyme 2 (ACE2) receptor [6]. The Gamma (P.1) and Delta (B.1.617.2) variants possess the highest replicative fitness in human airway epithelia, whereas the Omicron (BA.1) variant exhibits reduced replicative fitness and enhanced immune activation in the lower airway [7]. Subsequent Omicron sublineages have further evolved to demonstrate decreased pathogenicity [8]. Variants such as BQ.1.1 and XBB exhibit resistance to therapeutic monoclonal antibodies (REGN10987, REGN10933, COV2–2196, COV2–2130, S309 and LY-CoV1404) [9] while remaining susceptible to antiviral drugs (remdesivir, molnupiravir, and nirmatrelvir) [9,10]. Considering the ongoing evolution of SARS-CoV-2, understanding the dynamics of diverse viral fitness profiles is essential for assessing variant impacts, guiding antiviral drugs and vaccines development, and informing public health strategies for managing future pandemics [8,11–15].

Extensive efforts have been made to evaluate viral fitness. Experimentally, approaches such as deep mutational scanning (DMS) [16–22] and high-throughput in vitro evolution [23,24] have been developed to construct genotype–phenotype maps for ACE2 binding, cell entry, and antibody binding. These methods provide essential data for computational modeling. Machine learning frameworks based on these experimental data have been introduced [25–27]. For example, a language model was used to predict the immune escape of SARS-CoV-2 with specific mutations in the spike protein [28]. Deep learning methods involving multitask learning [29] and protein‒protein interactions [30] have expanded the range of antibodies for which escape potential can be predicted. Additionally, genomic surveillance data have facilitated the estimation of viral fitness, either through lineage classification [31], independent of lineage classification [32], or across different SARS-CoV-2 proteins [12]. These studies have provided critical insights into viral fitness; however, they have largely focused on overall viral fitness without characterizing the heterogeneity influenced by the immune contexts in which the virus evolves.

Viral fitness results from the complex interplay between intrinsic viral properties and population immunity [33]. The global rollout of COVID-19 vaccines has profoundly altered the immune landscape, with diverse vaccine platforms—including mRNA, adenovirus vector, and inactivated virus formulations—administered across different populations [34–38]. This heterogeneity, combined with varying histories of natural infection, has led to broad diversity in population immune backgrounds [2,39,40], which impose distinct selective pressures on viral evolution [41]. These selective pressures have facilitated the accumulation of adaptive mutations, which have further modulated the SARS-CoV-2 fitness profile [8,42–44]. However, the dynamics of SARS-CoV-2 fitness under diverse immune backgrounds and mutational landscapes remain poorly understood. Efforts to address this knowledge gap are essential for understanding the interplay between population-level immunity and viral evolution.

Addressing this gap requires computational models that capture synergistic effects of multiple mutations. Site-level scoring models based on DMS data remain a valuable tool for predicting immune escape of variants, yet their assumption of independent site contributions inherently limits their ability to capture epistatic interactions [45]. To this end, we developed a graph representation learning model, Geno-GNN, to predict ACE2 binding affinity and immune escape potential within diverse immune types solely based on viral sequence data. Unlike additive models, Geno-GNN learns complex non-linear dependencies among amino acids through the message-passing mechanism of a Graph Neural Network (GNN), thereby effectively integrating the impact of multiple mutations at the entire-variant level. In addition, we represented the protein as a heterogeneous graph with different types of nodes (amino acids) and edges (interactions), providing the model with richer and more biologically realistic relational information. By integrating extensive SARS-CoV-2 sequences from the Global Initiative on Sharing All Influenza Data (GISAID), Geno-GNN identified variations in SARS-CoV-2 fitness dynamics associated with heterogeneous immune backgrounds. Furthermore, through virtual mutation scanning, Geno-GNN revealed the fitness trajectories under diverse mutational landscapes. This retrospective analysis improves our understanding of viral fitness dynamics across diverse immune contexts and provides critical insights concerning the evolutionary trajectories of SARS-CoV-2 in real-world settings.

## Results

### Geno-GNN and its performance in predicting viral fitness

Geno-GNN was developed to predict the multiple fitness types by employing the amino acid sequence of the SARS-CoV-2 receptor-binding domain (RBD) as input (S1 Fig; see Methods for details). Fitness values were derived from DMS data, incorporating ACE2 binding affinity [43,46] and immune escape across five immunity types [18,19,42]: wild-type (WT) convalescent (WT infection), WT vaccine (three doses of CoronaVac), BA.1 + BTI convalescent (BA.1 infection postvaccination), BA.2 + BTI convalescent (BA.2 infection postvaccination), and BA.5 + BTI convalescent (BA.5 infection postvaccination) (S1 Table; Methods).

Geno-GNN demonstrated robust performance in predicting SARS-CoV-2 fitness. Tenfold cross-validation yielded a Spearman correlation coefficient of 0.95 for ACE2 binding affinity and showed reasonable predictive accuracy for immune escape, with correlation values ranging from 0.67 to 0.76 (S2A Fig). External validation further confirmed model reliability; Spearman correlation analysis produced correlation coefficients of 0.88 and 0.86 for ACE2 binding affinity across two independent test datasets (S2B–C Fig). Importantly, to quantify Geno-GNN’s ability to capture epistatic effects, we compared its performance against a naive additive model using the external dataset from Moulana et al. [23], which contains over 32,000 multi-mutation variants. Geno-GNN achieved a Spearman’s correlation of 0.86 (S2C Fig), substantially outperformed the additive model’s 0.66 (S2D Fig), providing direct evidence for its predictive power for variants with complex mutation combinations. For immune escape, predictions showed strong agreement with neutralization titers (Spearman correlation ranging from -0.64 to -0.86, S2E–H Fig). Notably, Geno-GNN outperformed a widely used site-independent escape calculator [45], which achieved only moderate from 0.40 to 0.66 on the same datasets (S3 Fig). Collectively, these results highlight Geno-GNN’s ability to capture epistatic interactions governing both ACE2 binding affinity and immune escape. At the site level, the predictions were highly accurate, with correlation coefficients of 0.99 for ACE2 binding affinity and 0.92–0.94 for immune escape across diverse immune types (S4A–F Fig). Comprehensive details are available in S1 Text.

### Temporal dynamics of viral fitness characterized by Geno-GNN

In total, we gathered 13,346,039 SARS-CoV-2 genomes from the GISAID database, covering the period from December 2019 to February 2024. Recognizing potential nonuniformity in genome surveillance capacity, we used a subsampling approach to ensure a balanced spatiotemporal distribution of genome sequences. Specifically, 200 sequences were randomly selected for each month and country; this procedure was repeated 100 times (Methods). These sequences were used to retrospectively assess the temporal dynamics of SARS-CoV-2 fitness at the population level via Geno-GNN.

**Fig 1:**
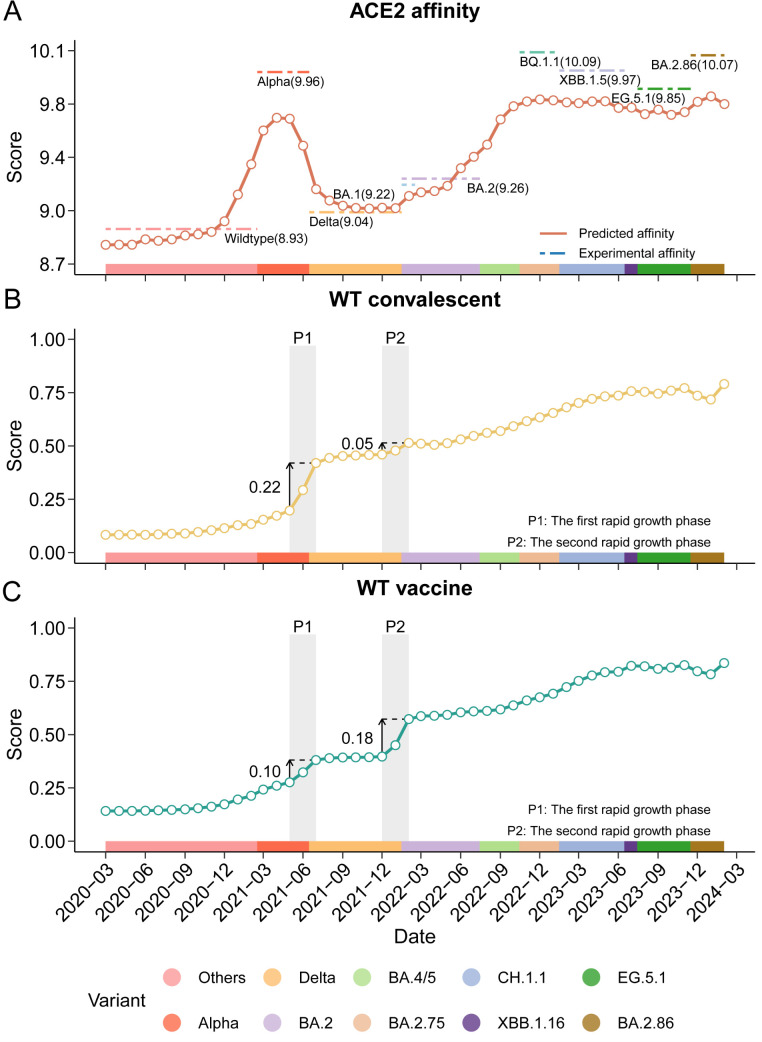
Temporal dynamics of SARS-CoV-2 fitness, as characterized by Geno-GNN. **(A)** Fluctuate-rise-stabilize pattern of angiotensin-converting enzyme 2 (ACE2) binding affinity. Horizontal colored dashed lines represent the experimental ACE2 binding affinity for each variant as measured by deep mutational scanning (DMS) [47,48] (values shown in parentheses). ACE2 binding affinities for variants BQ.1.1, XBB.1.5, EG.5.1 and BA.2.86 were used for external validation. **(B)** Stepwise pattern of immune escape against the wild-type (WT) convalescent immunity type. **(C)** Stepwise pattern of immune escape against the WT vaccine immunity type. The x-axis represents the monthly time points from March 2020 to February 2024, and the left y-axis represents the average fitness values of sampled sequences. Shaded gray regions highlight periods of rapid fitness increases, and corresponding fitness differences are indicated. Color gradients represent the predominant variant for each month, defined as the variant with the highest percentage in a given month (S5 Fig).

An initial phase of fluctuation in ACE2 affinity was observed, followed by a gradual increase and eventual stabilization (Fig 1A). This fluctuate-rise-stabilize pattern, which aligns with DMS experimental data [47,48] across diverse SARS-CoV-2 variants, may have resulted from the emergence and spread of successive variants of concern (S5 Fig). These dynamics likely reflect the interplay between short-term stochasticity and long-term directional trends in viral evolution [49].

A similar increasing trend in the ability of SARS-CoV-2 to evade both the WT convalescent and WT vaccine immunity was observed, characterized by a stepwise pattern consistent with the rising global burden of natural infections and expanding vaccine coverage (Fig 1B–C). Notably, two distinct phases of rapid growth in the escape score were identified for these immunity types, each displaying unique characteristics.

The first phase, corresponding to the 3-months period during which the dominant variant shifted from Alpha to Delta (May to July 2021), was characterized by a significantly greater increase in the escape score for the WT convalescent immunity type compared with that for the WT vaccine immunity type (change magnitude: 0.22 for WT convalescent, 0.10 for WT vaccine, *p* < 0.001, Wilcoxon test; Figs 1B–C and S6A). This observation suggests that SARS-CoV-2 was under substantial immune pressure from natural infections. In contrast, during the second phase (December 2021 to February 2022), when Omicron first emerged (December 2021) and subsequently replaced Delta as the dominant variant (January to February 2022), the magnitude of change for the WT vaccine immunity type exceeded that for the WT convalescent immunity type (change magnitude: 0.18 for WT vaccine, 0.05 for WT convalescent, *p* < 0.001; S6B Fig). This transition indicates a shift in immune pressure influenced by the combined effects of natural infections and increasing vaccine-induced immunity as vaccine coverage expanded.

The escape scores of SARS-CoV-2 from the BA.1 + BTI convalescent and BA.2 + BTI convalescent immunity types continued to increase only after BA.4/5 became the predominant epidemic variant (S7A–B Fig). No significant escape from the BA.5 + BTI convalescent immunity type was detected by Geno-GNN as of February 2024 (S7C Fig), consistent with previous findings [42,50]. Importantly, although the ability of pre-Omicron variants to escape sera from individuals who had recovered from Omicron infection is relevant, it does not reflect the immune pressure that drove viral evolution at that time.

### Heterogeneity in SARS-CoV-2 fitness across immune backgrounds revealed by Geno-GNN

To further explore the fitness dynamics of SARS-CoV-2, we performed clustering analysis based on fitness values across countries (Methods). Given that vaccines were not administered during the period dominated by variants categorized as “Others” and that the immune escape exhibited by Omicron reduced the efficacy of multiple vaccine types [19], we focused on the period after the predominance of “Others” variants and before the Omicron outbreak (December 2020–January 2022, S5 Fig). We identified two main clusters in the data (S8A Fig). Intriguingly, these clusters showed a clear alignment with the immune backgrounds categorized by the administered vaccine platforms (S8B Fig). The larger cluster primarily consisted of countries where mRNA vaccines were administered, whereas the smaller cluster predominantly included countries where most vaccines were inactivated or adenovirus vector-based.

To investigate whether the observed pattern was driven by confounding factors, we employed a multiple linear regression model controlling for monthly new cases, the natural immunity coverage (adjusted for reinfection protection), vaccine-induced immunity coverage (adjusted for immune waning), non-pharmaceutical intervention (NPI) stringency, the prevalence of major variants, and geographical sub-regions. Even after adjustment, vaccine type remained statistically significant predictor of all fitness phenotypes (S2 Table), indicating that the clustering in S8A Fig reflects an association between vaccine platforms and distinct viral fitness landscape. However, given its relatively modest effect size compared to other explanatory variables, vaccine type should be regarded as one of several contributing factors rather than the primary driver. Although no significant differences in overall viral fitness values were detected with respect to immune backgrounds (S9 Fig), these findings suggest that the adaptation of SARS-CoV-2 fitness varies according to the prevailing immune background.

**Fig 2:**
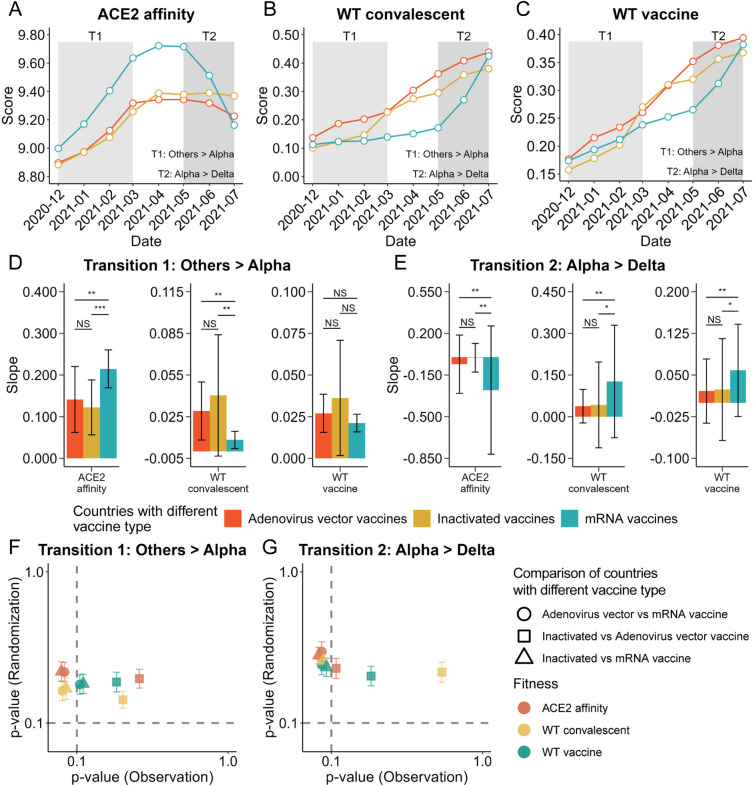
Heterogeneity in SARS-CoV-2 fitness dynamics across immune backgrounds. **(A–C)** Temporal trends in angiotensin-converting enzyme 2 (ACE2) binding affinity (**A**) and immune escape against the wild-type (WT) convalescent (**B**) and WT vaccine (**C**) backgrounds, stratified by vaccine type. Shaded regions indicate variant transition periods: T1 (Others-to-Alpha) and T2 (Alpha-to-Delta). **(D, E)** Fitness change rates across regions with different vaccine backgrounds during the Others-to-Alpha (**D**) and Alpha-to-Delta (**E**) transition periods. The y-axis represents estimated slopes (i.e., fitness change rates) with 95% confidence intervals (black error bars). *p* values are indicated: *p < 0.10; **p < 0.05; ***p < 0.01; ****p < 0.001; NS, not significant. **(F, G)** Statistical validation of the associations between fitness change rates and immune backgrounds during the Others-to-Alpha (**F**) and Alpha-to-Delta (**G**) transition periods. The x-axis represents *p* values from the observed slope comparisons, whereas the y-axis represents mean *p* values from 100 random permutations with 95% confidence intervals. Shapes correspond to the compared immune backgrounds, and colors indicate fitness types. Horizontal and vertical dashed lines indicate the significance threshold (0.1).

To investigate variations in SARS-CoV-2 fitness, we performed immune-background-specific piecewise regression analysis, stratified by the dominance and transition periods of the predominant variants (Fig 2A–C, S3 Table, Methods). Regions utilizing mixed vaccines were excluded because of the complexity of their immune backgrounds. The analysis revealed distinct fitness change rates according to immune background (Methods, S4 Table).

During the “Others-to-Alpha” transition period, regions with an mRNA vaccine immune background exhibited a greater change in ACE2 binding affinity relative to those with adenovirus vector- or inactivated-vaccine immune backgrounds {change rate: 0.21 [95% confidence interval (CI): 0.17–0.26] for mRNA-vaccinated regions, 0.14 [95% CI: 0.06–0.22] for adenovirus vector-vaccine regions, and 0.12 [95% CI: 0.06–0.19] for inactivated-vaccine regions; Fig 2D, S3 Table}. Conversely, regions with adenovirus vector and inactivated vaccine backgrounds exhibited faster changes in immune escape against the WT convalescent and WT vaccine immunity types relative to regions with mRNA vaccine backgrounds (Fig 2D). This finding suggests a trade-off between the escape score and ACE2 affinity; this trade-off differed according to immune context.

During the “Alpha-to-Delta” transition period, regions with an mRNA vaccine immune background also exhibited faster changes in both ACE2 binding affinity and immune escape relative to those with WT convalescent and WT vaccine backgrounds. Moreover, regions with adenovirus vector- and inactivated-vaccine immune backgrounds displayed a more balanced pattern involving gradual changes in fitness (Fig 2E). To assess the effect of immune background on fitness change rates, 100 random permutations of vaccine assignments were performed while maintaining their original distributions. The resulting *p* values did not show statistically significant differences in change rates across comparisons (Fig 2F,2G), indicating that the observed heterogeneity was unlikely to have occurred by chance and suggesting that the immune background influences SARS-CoV-2 fitness dynamics. Multiple linear regression, controlling for geographical sub-regions, major variant prevalence, and epidemiological metrics, further confirmed that vaccine type influenced fitness change rate: during the “Others-to-Alpha” transition period, it was a significant predictor for all three phenotypes (all *p*-values < 0.001; S5 Table), while during the ‘Alpha-to-Delta’ period, its effect remained significant for ACE2 affinity (*p* < 0.001) and WT convalescent escape (*p* < 0.001) but not for WT vaccine escape (*p* = 0.778; S6 Table).

During the dominance periods of the predominant variants, differences in fitness change rates were also observed between regions with an mRNA vaccine immune background and those with adenovirus vector- or inactivated-vaccine immune backgrounds (S3 and S4 Tables). However, change rates in regions with adenovirus vector- or inactivated-vaccine immune backgrounds were generally similar, consistent with the clustering results shown in S8A Fig. These immune-background-specific differences highlight the complex interactions between intrinsic viral properties and immune contexts. However, the underlying mechanisms for these disparities remain unclear, and no definitive conclusions have been reached.

### Geno-GNN-based exploration of SARS-CoV-2 fitness trajectory

**Fig 3:**
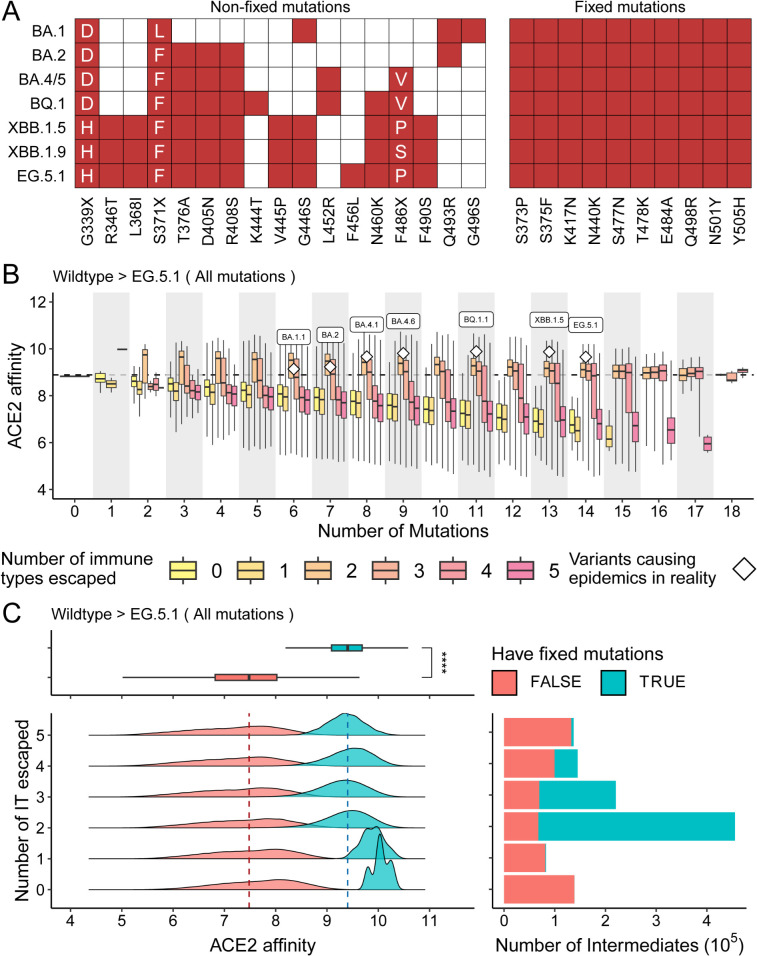
SARS-CoV-2 fitness trajectory, as revealed by Geno-GNN. **(A)** Distribution of RBD mutations across major epidemic variants, including BA.1, BA.2, BA.4/5, BQ.1, XBB.1.5, XBB.1.9, and EG.5.1. Fixed mutations, shared by multiple variants, are highlighted. The x-axis represents mutation types; for loci with multiple mutations, specific types are displayed in the corresponding cell. **(B)** Fitness trajectories of SARS-CoV-2 identified through virtual mutation scanning. ACE2 binding affinities are grouped by the number of mutations and the number of immune types that escaped. Major real-world variants are denoted by white rhombuses. Color gradient represents the number of escaped immune types. Black dashed line indicates the ACE2 affinity of the wild type. “All mutations” refers to the complete set of RBD mutations in major variants relative to the wild-type strain, defining the combinatorial mutation space. Fixed mutations are regarded as a single mutation and assumed to co-occur or co-disappear in virtual mutation scanning. **(C)** Differentiation of two distinct trajectories based on fixed mutations. ACE2 binding affinities are categorized by the number of immune types escaped (IT: immune type). Median ACE2 affinities for intermediates with and without fixed mutations are indicated by blue and red dashed lines, respectively. Bar chart presents the number of intermediates in each immune type. Boxplots compare ACE2 affinities for intermediates with and without fixed mutations; statistical significance is annotated. Boxes represent the interquartile range (25th–75th percentiles), and whiskers extend to 1.5 times the interquartile range. Fixed mutations are shown in color.

To retrospectively investigate the fitness trajectory of SARS-CoV-2 under mutational landscapes, we performed virtual mutation scanning analysis. Specifically, all possible pseudovirus RBD sequences were simulated, spanning from Wuhan-Hu-1 to sequences that contained all RBD mutations observed in major epidemic variants: BA.1, BA.2, BA.4/5, BQ.1, XBB.1.5, XBB.1.9, and EG.5.1. To simplify the combinatorial mutation space, fixed mutations—defined as those shared across all studied variants—were co-mutated during virtual mutation scanning (Fig 3A, Methods). In total, 1,179,648 RBD intermediates were generated, and the corresponding fitness values were evaluated using Geno-GNN.

When stratified by the number of mutations and the number of immune types escaped, two distinct fitness trajectories were observed (Fig 3B). One trajectory involved increasing immune escape as mutations accumulated, ultimately leading to the escape of all immune types at the expense of ACE2 affinity. In contrast, the other trajectory maintained ACE2 affinity at or above the Wuhan-Hu-1 level while escaping a moderate number of immune types (typically 2–3). Notably, SARS-CoV-2 variants associated with major outbreaks in real-world scenarios predominantly followed the latter trajectory, indicating negative selection against variants in the former trajectory during the evolutionary process. These findings suggest an evolutionary pathway that contributes to the emergence of variants in real-world epidemics, highlighting the critical roles of ACE2 binding affinity and immune escape capacity in the adaptive landscape of SARS-CoV-2.

Further analysis of these intermediates revealed that fixed mutations may drive the divergence of these two trajectories. Mutations were predominantly observed in the latter trajectory, maintaining a higher ACE2 affinity (median ACE2 affinity: 9.40 for variants with fixed mutations versus 7.48 for those without; *p* < 0.001, Wilcoxon test; Fig 3C). Additionally, intermediates without fixed mutations were relatively evenly distributed across different immune types, whereas most intermediates with fixed mutations escaped 2–4 immune types (Fig 3C). These findings emphasize the central role of fixed mutations in SARS-CoV-2 evolution.

### Fitness effects of mutations quantified by Geno-GNN

Building on the fitness characterization of the mutation space, we investigated the contributions of individual mutations to SARS-CoV-2 fitness within the combinatorial mutation landscape. Specifically, we assessed the impact of each mutation by comparing the ACE2 affinities of intermediates with and without the mutation. The contribution of each mutation to immune escape was determined by the percentage of intermediates carrying the mutation and escaping a given immune type relative to all intermediates (Methods). In fitness trajectory analysis, fixed mutations were considered co-mutated during virtual mutation scanning. To evaluate the impact of each individual fixed mutation on fitness, a separate virtual mutation scanning analysis was performed, considering each fixed mutation separately (S10 Fig).

**Fig 4:**
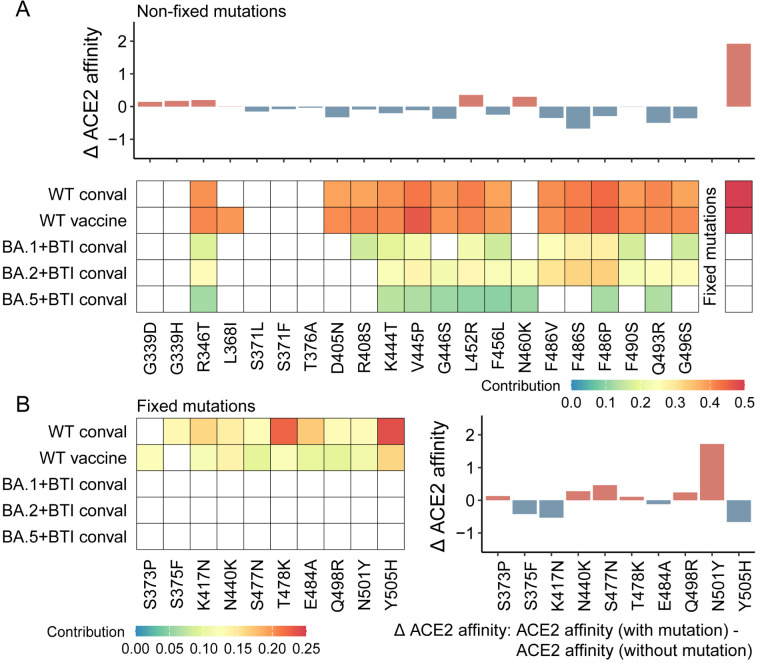
Quantification of the fitness effects of mutations in major circulating variants within the combinatorial mutation landscape, according to Geno-GNN. **(A)** Contributions of nonfixed mutations to ACE2 affinity (upper panel) and immune escape (lower panel, conval: convalescent). **(B)** Contributions of fixed mutations to ACE2 affinity (right panel) and immune escape (left panel). Heatmap color scale indicates the extent of immune escape attributed to each mutation. White represents the absence of a contribution to immune escape for a given immune type. In bar plots, red bars indicate an increase in ACE2 affinity, whereas blue bars indicate a decrease. “Δ ACE2 affinity” represents the difference in the median ACE2 binding affinity between receptor binding domain (RBD) sequences with and without the mutation.

Nonfixed mutations generally reduce ACE2 binding affinity but exhibit diverse immune escape potentials (Fig 4A). In contrast, fixed mutations significantly increase ACE2 affinity, particularly the compensatory mutation N501Y [23]. These fixed mutations also contribute to immune escape, primarily by facilitating escape from WT convalescent and WT vaccine immunity (Fig 4A–B). These findings suggest a complementary relationship between fixed and nonfixed mutations in shaping viral adaptability, with fixed mutations enhancing ACE2 affinity while nonfixed mutations primarily drive immune escape.

At the individual mutation level, certain nonfixed mutations resulting from convergent evolution [42]—such as R346T, K444T, V445P, G446S, L452R, N460K, F486V, F486P, F486S, and F490S—exhibit substantial and broad contributions to immune escape, albeit with variable effects on ACE2 binding affinity. Conversely, fixed mutations, such as K417N, T478K, E484A, and Y505H, strongly contribute to immune escape, although most of these (except T478K) tend to weaken ACE2 binding affinity. Our results are consistent with previous findings [19,51–54]. BA.2.86, the last variant to emerge during the study period, retains all the fixed mutations except E484A, which is replaced by E484K, further confirming the key role of fixed mutations in viral fitness evolution (S7 Table). These findings highlight the complex roles of mutations in balancing immune escape and ACE2 binding affinity.

## Discussion

While the COVID-19 pandemic phase has officially ended, SARS-CoV-2 continues to spread and evolve [55], underscoring the ongoing need for timely evaluation of its various fitness types. Such assessments are crucial for guiding COVID-19 vaccine updates, managing infectious diseases, and increasing our understanding of virus evolution. In this study, we developed a graph representation learning model to evaluate multiple SARS-CoV-2 fitness types on the basis of RBD variants and revealed fitness dynamics heterogeneity across immune backgrounds and possible fitness trajectories under varying mutational landscapes. Our results highlight the utility of the graph representation learning model in elucidating the population-level fitness dynamics of virus.

The extensive data generated by DMS experiments offer a valuable opportunity to model the functional impacts of SARS-CoV-2 mutations, particularly their ability to bind ACE2 and escape from diverse immunity types. A simple transformation of direct DMS experimental measurements was proposed to estimate the antigenic effects of mutations at the site level [45], offering a useful framework. However, this approach may not fully capture potential variations in the effects of different types of mutations at the same site. Additionally, it assumes that the impact of mutations on escape is independent across different sites, which may not always reflect the complexity of mutation interactions. In contrast, our Geno-GNN model can leverage information from mutations across the entire RBD, allowing for mutation-level assessment that better capture the complex correlations among mutations, thereby offering more practical insights. In addition, earlier deep learning models [29,30] primarily assessed escape potential at the level of individual antibodies, whereas Geno-GNN evaluates the escape potential against human sera with diverse immunity types. This broader perspective increases the applicability of Geno-GNN to real-world scenarios and offers deeper insights into viral fitness dynamics at the population level.

Notably, in ACE2 affinity prediction, Geno-GNN performed well in ten-fold cross-validation (*R* = 0.95) and slightly lower on two external test sets (0.88 and 0.86). This gap likely reflects intrinsic difference in mutational complexity across the datasets. The training data mainly contained low-order mutations, whereas the external test sets included numerous high-order variants with over 15 mutations. Predicting such variants, shaped by complex non-additive epistatic effects, is inherently challenging. Thus, the modest performance decline observed for Geno-GNN on unseen high-order mutations supports its strong generalization ability and robustness.

With the advancement of high-throughput genome sequencing, characterizing viral genetic changes over time has become increasingly feasible. A previous study revealed a boom-and-bust pattern in the genetic diversity of influenza A (H3N2), which was associated with antigenic cluster transition [56]. For SARS-CoV-2, genomic diversity has exhibited punctuated evolution over time [57]. However, capturing the temporal dynamics of viral fitness remains a significant challenge. In this study, by exploiting our Geno-GNN model, we identified a fluctuating, stable pattern in ACE2 binding affinity that correlates with SARS-CoV-2 variant transitions. Additionally, we observed a stepwise increase in the ability of the virus to escape immunity derived from both the WT convalescent and WT vaccine backgrounds. This finding complements previous observations of a steady increase in escape scores based on a single-antibody response [30], highlighting the dynamic nature of the evolutionary fitness of SARS-CoV-2. Meanwhile, we observed variations in the magnitude of the rapid fitness growth phase as SARS-CoV-2 escaped WT convalescent immunity type and WT vaccine immunity type, suggesting a shift in the sources of immune pressure.

The widespread use of COVID-19 vaccines has shaped diverse immune profiles within populations [2,39]. Leveraging real-world SARS-CoV-2 sequences, our study reveals that viral fitness exhibits distinct clustering patterns in regions utilizing different types of vaccines. Additionally, during variant transition periods, these regions displayed significant differences in fitness change rates, reflecting a trade-off between immune escape and ACE2 affinity. Although previous studies reported the trade-off [25,30,58], our research further uncover that it manifested in diverse ways across different populations with varying types of COVID-19 vaccines in use. While the underlying drivers of this heterogeneity remain unclear, our findings underscore the nonuniform and complex evolution of SARS-CoV-2 across diverse immune backgrounds.

Moreover, virtual mutation scanning analysis revealed that real-world SARS-CoV-2 variants may follow a fitness trajectory that maintains ACE2 affinity at or above the Wuhan-Hu-1 level while a moderate number of immune types are escaped. Fixed mutations appear to compensate for ACE2 affinity and a baseline level of immune escape [19,23,51–54,59]. Moreover, we observed that although certain mutation combinations could theoretically escape from all immune types, variants carrying these combinations along with fixed mutations rarely achieve complete immune escape. This result suggests that, after the acquisition of fixed mutations, variants may require additional escape mutations to increase their immune escape capacity. These findings expand upon those of earlier work [58] that was focused on monoclonal antibodies by encompassing broader, real-world immune types.

The seminal work by Hie et al. [28] conceptualized escape mutations as analogous to words that change “semantics” while maintaining “grammaticality”—that is, altering the virus’s immune signature while preserving its infectivity. Our analysis suggests that the evolutionary trajectory of SARS-CoV-2 generally adheres to this principle. Importantly, our analysis indicates that this delicate balance appears to result from the synergistic interaction among multiple mutations under evolutionary selection, not by isolated mutations, analogous to modifying several words in a sentence to preserve grammatical fluency while reconstructing the underlying semantic structure. This refined interpretation—from the function of individual “words” to the synergistic grammar of “word” combinations—offers deeper understanding of viral evolution.

There are several limitations to our study. Although a few thousand antibodies were evaluated on the basis of DMS experiments, this method still may not fully capture the complexity of human sera. Additionally, our analysis was confined to the RBD of the spike protein because of the availability of ACE2 binding and immune escape data. Future research will expand on this by utilizing an updated DMS platform that targets the full SARS-CoV-2 spike protein [20,21]. While we explored five different immune types, incorporating DMS data from mRNA COVID-19 vaccines before Omicron outbreaks would provide a more comprehensive view in our analysis. The immune backgrounds in our study were defined on the basis of the administered vaccine platforms. Although some studies have shown distinct immune responses among different types of vaccines [60], natural infections may also contribute to the immune background [61]. More importantly, as a retrospective study, our research does not aim to predict which newly emerged strains or unobserved mutations are most likely to spread widely. Instead, we focus on evaluating the current fitness landscape based on observed sequences, offering insights into the population-level dynamics of the virus. The disparities in sequence submissions across countries were mitigated on the basis of carefully designed sampling. Despite the unavoidable impact of cross-border transmission, we reduced its impact by merging countries and still observed heterogeneity in viral fitness, although the underlying causes remain unclear. The significant decline in globally submitted sequences on GISAID after September 2022 may increase uncertainty regarding the dynamics of viral fitness. However, this decline should not alter the overall pattern. Owing to computational constraints, we did not capture all the potential fitness trajectories of SARS-CoV-2 with our virtual mutation scanning; however, DMS did reveal the fitness trajectory of variants that may have driven real-world epidemics.

In summary, our study provides a comprehensive analysis of SARS-CoV-2 fitness dynamics through the application of our graph representation learning model, Geno-GNN. This model provides insights into the associations between variations in SARS-CoV-2 fitness dynamics and immune backgrounds, as well as fitness trajectories. These findings offer valuable insights into the complex evolution of virus and its fitness dynamics at the population level.

## Materials and methods

### Definition of fitness and fitness dynamics

In viral evolution, fitness is the virus’s ability to replicate and transmit within a host population under given conditions, typically inferred from measurable phenotypes rather than measured directly. Here, we define *fitness* as a multidimensional phenotypic profile. This profile consists of two core components: first, the ACE2 affinity, which reflects the efficiency of viral entry into cells; and second, the immune evasion capability under five immunity types: wild-type (WT) convalescent (WT infection), WT vaccine (three doses of CoronaVac), BA.1 + BTI convalescent (BA.1 infection postvaccination), BA.2 + BTI convalescent (BA.2 infection postvaccination), and BA.5 + BTI convalescent (BA.5 infection postvaccination). *Fitness dynamics* refers to the temporal trajectory of change in the six fitness components.

### Fitness data collection and processing

The impacts of SARS-CoV-2 RBD mutations on ACE2 binding affinity were sourced from the research conducted by Starr *et al.* [43,46]. Their dataset encompasses both single and multiple mutations within the RBDs of seven SARS-CoV-2 variants (Wuhan-Hu-1, Alpha, Beta, Delta, Eta, BA.1 and BA.2) and the corresponding ACE2 binding affinity values, which are quantified with respect to the apparent dissociation constant (K_D,app_) measurements via the DMS technique. To provide the model with sufficient information and avoid overfitting, we included all single mutation data from the dataset and selected double mutation data on the basis of the following criteria: the difference between the fitness value of the double mutation and the sum of the fitness values of its two single mutations is greater than 0.1. This approach ensures that the double mutation data capture the nonlinear changes in ACE2 affinity caused by combinatorial mutations while avoiding redundant usage of single mutation information.

Data on the antibody escape capacity of SARS-CoV-2 variants were compiled from Cao’s experiments [18,19,42]. In these experiments, the escape scores of SARS-CoV-2 RBD mutations against monoclonal antibodies found in serum from populations of different immune types were determined. The escape scores were determined by comparing the frequency of variant barcodes in immune-escaping cell populations to the frequency of variant barcodes in the reference population and then normalizing with respect to each antibody. On the basis of the source of immunity, we categorized these monoclonal antibodies into five groups: individuals who recovered from the original variant; those who recovered from the BA.1, BA.2, and BA.5 variants; and individuals vaccinated with three doses of inactivated vaccines. After removing duplicate antibodies, escape scores for each mutation (amino acid level) at a single site were averaged within the same immunity group, representing the effect of this mutation on the corresponding immune type. We assumed that averaging escape score across antibodies with the same immunity group would effectively represent the characteristics of actual human sera, a hypothesis used in previous study [45].

### Implementation and training of the Geno-GNN model

For Geno-GNN, the input consists of the 201-amino acid sequence of the SARS-CoV-2 RBD. The properties of each amino acid are characterized using VHSE, which stands for Vectors of Hydrophobic, Steric, and Electronic properties—a set of amino acid descriptors derived from principal component analysis of 18 types of hydrophobic, 17 types of spatial, and 15 types of electronic properties [62]. Each amino acid in the RBD sequence is represented by an 8-dimensional numerical vector composed of these principal components. These numerical vectors, which represent every residue in the sequence, are concatenated to depict an RBD variant. In summary, we structured the input sequence data as a 201ⅹ8 matrix based on VHSE and organized it in sequential order to form an undirected graph for subsequent predictions.

Inspired by inductive representation learning on large graphs [63] and the effectiveness of the global pooling operation in processing the structured data [64], our Geno-GNN model consists of three components: a convolution module, a global pooling layer, and three fully connected layers. The use of GNN is motivated by its ability to capture the complex dependencies between amino acids, particularly by considering the sequential relationships within the RBD. Through neighborhood aggregation, GNN can model both local and global information of the RBD variants, thereby enhancing the accuracy of predictions.

Our Geno-GNN model framework is composed of three modules connected in series: a graph convolution module, a global pooling module, and a prediction head module for the final prediction.

For the convolution module, a 2-layer Relational Graph Convolutional Network (R-GCN) model [65] is adopted to encode the node (amino acid) features and learn their embeddings. R-GCN is specifically designed for heterogeneous graphs and can effectively handle the complex graph structure we constructed, which contains up to 20 node types (for the 20 amino acids) and 420 edge types (including 400 pairwise node combinations and 20 self-loop edges). Its core layer-wise update rule is as follows:


$$\begin{array}{c} h_{i}^{\left(l+\text{1}\right)}=\text{ReLU}(\sum {\mathrm{\backslash nolimits}}_{r\in R}\sum {\mathrm{\backslash nolimits}}_{j\in N_{i}^{r}}\frac{\text{1}}{c_{i,r}}W_{r}^{\left(l\right)}h_{j}^{\left(l\right)}+W_{\text{0}}^{\left(l\right)}h_{i}^{\left(l\right)}) \end{array}$$

In this formula, $h_{i}^{\left(l\right)}$ represents the embedding vector of node i at layer l, R is the set of all relation types, and $N_{i}^{r}$ is the set of neighboring nodes of node i under relation r. $W_{r}^{\left(l\right)}$ and $W_{\text{0}}^{\left(l\right)}$ are the learnable weight metrices for each relation r and for self-loops, respectively, while $c_{i,r}$ is a normalization constant, set to $\left|N_{i}^{r}\right|$ to balance the information aggregation from neighbors. After two convolutional layers (with a hidden layer dimension of 256), the model generates as a final 256-dimensional embedding vector for each amino acid in the RBD sequence. All these vectors constitute the embedding matrix $H\in R^{n\times \text{2}\text{5}\text{6}}$, where n denotes the total number of amino acids in the RBD sequence.

Next, the global pooling module aggregates these node-level embeddings into a single graph-level representation. In our model, we employ a Concatenation operation that directly links all final node embedding vectors $h_{i}$, followed by a Flatten operation to form a one-dimensional graph representation vector Z.


$$Z=\text{Flatten}(\text{CONCAT}(h_{\text{1}},h_{\text{2}},\cdots ,h_{n}))$$

This operation generates a graph vector with a dimension of 1×51456, which has the advantage of completely preserving the embedding information from all amino acid nodes in the sequence.

Finally, this graph representation vector Z is fed into a 3-layer fully connected network (i.e., a Multi-Layer Perceptron, MLP), which serves as the prediction head, to compute the final regression prediction value $\hat{y}$. The computational process of this network is as follows:


$$h_{\text{MLP1}}=\text{ReLU}(ZW_{\text{MLP1}}+b_{\text{1}})$$

$$h_{\text{MLP2}}=\text{ReLU}(h_{\text{MLP1}}W_{\text{MLP2}}+b_{\text{2}})$$

$$\hat{y}=h_{\text{MLP2}}W_{\text{MLP3}}+b_{\text{3}}$$

Here, W and b represent the weight matrices and bias terms of each layer, respectively. The output dimensions of the two hidden layers are set to 64 and 32, respectively, while the final output layer has a dimension of 1. The activation function for all layers except the output layer is ReLU, and no Dropout regularization is used in the model. The entire Geno-GNN model is trained end-to-end using undirected graphs representing RBD variants and their corresponding fitness data.

The Geno-GNN model, which was implemented using PyTorch, was trained for 500 epochs by utilizing the Adam optimizer with a learning rate of 3e^−5^. To prevent overfitting, we applied an early stopping strategy in the training process. When the *R*^2^ of the validation set does not increase over 20 consecutive training epochs, the training is terminated early to avoid unnecessary computations and protect the model’s generalization ability. The reported prediction accuracies were obtained by aggregating the outputs of tenfold cross-validation. For ACE2 affinity, a dataset comprising all single mutations and a randomly selected 10% of double mutations was utilized for training and validation. Specifically, 80% of the data from each of the single mutations and double mutations were allocated for training, with the remaining 20% of each reserved for validation, and this process was repeated through ten-fold cross-validation. Additionally, 90% of the double mutations from the original dataset were designated for testing, ensuring a robust evaluation of the model’s performance. For immune escape, 10% of the dataset was randomly partitioned for testing, while the remaining data were divided into ten subsets of equal size for cross-validation. The hyperparameters described above are selected from multiple experiments to obtain the optimal results. In total, six models were trained to correspond with the six fitness types discussed in this study.

### External validation dataset

For ACE2 binding affinity analysis, complementary external validation data were obtained from the studies of Taylor *et al*. [47,48] and Moulana *et al*. [23]. Taylor *et al*. provided additional binding affinity data for all single mutations across four Omicron subvariants (BQ.1.1, XBB.1.5, EG.5, BA.2.86). Moulana *et al*. systematically evaluated the ACE2 binding affinity across all the feasible combinations for 15 mutations (2^15^ = 32,768 RBD variants) within the RBD of the BA.1 variant compared with the original Wuhan Hu-1. For immune escape analysis, the neutralization assay data generated by Cao *et al*. [42] were employed. This dataset includes pseudovirus neutralizing titers against Omicron subvariants from plasma samples of individuals who had received three doses of CoronaVac, individuals who had been infected with BA.1 after receiving three doses of CoronaVac, individuals who had been infected with BA.2 after receiving three doses of CoronaVac, and individuals who had been infected with BA.5 after receiving three doses of CoronaVac.

### Data sources for epidemiological and immunological analyses

As of February 28, 2024, we had collected 13,346,039 SARS-CoV-2 sequences with metadata from GISAID [66]. After applying the same quality control procedures as those used in the previous study [67], we distilled this dataset to 5,604,530 sequences suitable for in-depth analysis. The daily number of new COVID-19 cases was obtained from Our World Data [68]. The daily stringency index, which reflects the strictness of non-pharmaceutical interventions (NPIs), was extracted from the Oxford Coronavirus Government Response Tracker [69]. Furthermore, to account for population-level immunity, we incorporated two key immunological metrics described in a previous study [67]. Specifically, we utilized the calculated monthly natural immunity coverage, which was estimated based on cumulative infections from IHME and adjusted for the effectiveness of preventing reinfections. We also included the vaccine coverage metric, which was adjusted for waning immunity based on vaccine effectiveness against infection. Information regarding the types of COVID-19 vaccines administered in different countries was also gathered to serve as a basis for national classification [67].

### Temporal fitness pattern analysis

We performed a retrospective study of real-world SARS-CoV-2 sequence data. Our approach involved monthly sampling of 200 sequences from each country (if fewer than 200 sequences were available, all available sequences were extracted) to maintain an equal number of sequences per month across countries as much as possible. This sampling strategy was repeated 100 times to minimize the randomness of the samples. The sampled sequences were then fed into our Geno-GNN model for the assessment of their various fitness types. We averaged the fitness values from each country’s monthly samples and then further averaged these values across the 100 samples to chart the fitness dynamics. Additionally, we assessed the monthly prevalence of each major SARS-CoV-2 variant by analyzing our sampling data, and we defined each month as the epidemic period of the variant with the highest prevalence for that month. We used the Wilcoxon test to examine the magnitude of changes in the WT convalescent and WT vaccine groups during the two rapidly increasing periods across 100 samples.

### Clustering analysis for temporal variations in fitness

We utilized hierarchical clustering to cluster countries on the basis of the temporal variations in different fitness types. We first selected the fitness values for the period to be analyzed and normalized them for each fitness type. We then applied the average linkage method for clustering, and we employed the Euclidean distance as the distance between clusters. The average linkage method is used to calculate the distance between two clusters by taking the average distance of all pairs of points within the clusters.

### Detection of heterogeneity in the fitness change rate across immune backgrounds

We calculated the percentage of each variant using sequences from each sampling and then aggregated these percentages from 100 samplings to determine the monthly prevalence of each variant. The study period was then divided into different segments on the basis of the following criteria: (1) Dominant variant: The variant with the highest prevalence in a given month was designated the dominant variant for that month. (2) Variant transition period: This period begins when the prevalence of a new variant exceeds 5% and ends when it becomes the dominant variant. (3) Variant dominance period: This is the duration during which a variant consistently remains the dominant variant, excluding its transition period. On the basis of the above designations, eight distinct segments were obtained: the Others dominance period, the Others-to-Alpha transition period, the Alpha dominance period, the Alpha-to-Delta transition period, the Delta dominance period, the Delta-to-Omicron transition period, and the Omicron dominance period.

Countries were classified into four immune backgrounds on the basis of vaccine usage, which is consistent with a previous study [67]: regions where mRNA vaccines are administered, regions where inactivated vaccines are administered, regions where adenovirus vector vaccines are administered, and regions where mixed vaccines are administered. Owing to the complexity of the immunity types used in regions with mixed vaccines, only the first three categories were included in our analysis. From the sampling results, we extracted countries from these three region types to access SARS-CoV-2 fitness variations. Pairwise Wilcoxon tests were performed to assess the differences in the median fitness values across different immune backgrounds.

According to the defined periods and immune backgrounds, we conducted piecewise regression for each fitness type. Differences in regression coefficients (i.e., fitness change rates) between immune backgrounds across the specified periods were assessed via the slope difference test. We also performed 100 random permutations by reassigning vaccine types to individual countries, ensuring that the overall distribution of countries across vaccine categories remained consistent with the actual data. For each permutation, we conducted slope difference tests to assess differences in fitness change rates across immune backgrounds.

### Statistical analysis to control for confounding factors

To assess the impact of vaccine type on viral fitness, we implemented a series of multiple linear regression models incorporating various immunological and epidemiological factors as confounding variables. The analyses involved two main categories of models, with either (1) the fitness values or (2) the temporal rate of change in these fitness values as the dependent variable. Each model followed the general form:


$$Y=\;\beta_{\text{0}}+\beta_{\text{1}}X_{\text{1}}+\beta_{\text{2}}X_{\text{2}}+\cdots +\beta_{k}X_{k}+\epsilon$$

where Y is the dependent variable, $\beta_{\text{0}}$ is the intercept, $\beta_{\text{1}},..,\beta_{k}$ are regression coefficients, and ϵ is the error term. The variables $X_{\text{1}},\ldots ,X_{k}$ included were: adjusted natural immunity coverage and adjusted vaccine-induced immunity coverage; a binary variable for vaccine type (mRNA vs. non-mRNA); continuous variables representing the prevalence of major variants of concern (Alpha, Beta, Delta, Gamma); categorical dummy variables for UN geographical sub-regions; the stringency index of non-pharmaceutical interventions; and monthly new cases as a proxy for transmission intensity. All data were aggregated and aligned at the country-month level. Statistical significance for each variable was determined from by the *p*-value of its corresponding regression coefficient with a significance threshold of *p* < 0.05.

### Geno-GNN-based SARS-CoV-2 fitness trajectory identification

We applied Geno-GNN to predict fitness values for computer-generated synthetic viruses to explore possible fitness trajectories. Focusing on the major variants (BA.1, BA.2, BA.4/5, XBB.1.5, XBB.1.9, BQ.1 and EG.5.1), we searched for variants on the CoV-Spectrum website [70] by adding “(Nextclade)” after the variant names, with a time range from March 2020 to February 2024. From each search result, we selected mutations with a percentage greater than 90% to ensure their robustness. To proceed with variant generation, we compiled a union set of mutations (n = 31) from all the variants and categorized the mutations shared by all the variants as fixed mutations (n = 10), which were comutated during generation. Ideally, each site can have 19 possible mutations, resulting in 20 possible mutation scenarios. However, in our study, we considered only those mutations observed in real-world variants. The generation method was as follows: for a site with k (0 ≤ k ≤ 3, k ∈ N) observed mutations, the possible mutation scenarios are k + 1—either no mutation or mutation to one of the k mutations. The possible scenarios for different mutation sites are as follows: A single mutation site (e.g., site 452) has two possible mutation scenarios, and there are 15 such sites in the set, corresponding to 2^15^ possible mutation scenarios. A site with two mutations (e.g., site 339) has three possible mutation scenarios, and there are 2 sites in the set, corresponding to 3^2^ possible mutation scenarios. A site with three mutations (e.g., site 371) has 4 possible mutation scenarios. Finally, we generated all possible intermediates of the RBD sequence within the union mutation set, resulting in a total variant search space of 1,179,648 (n = 2^15^ × 3^2^ × 4), and predicted their fitness values using our models. We defined a variant as escaping a given immunity type if the corresponding escape score was higher than 0.5.

### Fitness effects of mutation quantification

We defined the contribution of a mutation to the immune escape type as follows: (1) For immune type *i* and mutation *m*, we calculated *p*1, the percentage of variants carrying mutation *m* among those escaping immune type *i*. (2) We calculated *p*2, the percentage of variants escaping immune type, *i*, relative to the total number of variants. If p1 ≤50% or p2≤5%, the mutation was considered noncontributory, and further calculation was terminated. (3) The contribution of mutation *m* to immune type *i* was determined as *p*1ⅹ*p*2, which represents the percentage of variants carrying mutation *m* and escaping immune type *i* among all variants.

## Supporting information

S1 Text The framework of Geno-GNN and its performance for six types of virus fitness. (DOCX)

S1 Fig Overview of Geno-GNN framework. We collected RBD variant sequences from deep mutational scanning data along with their corresponding ACE2 affinity and their ability to escape various immune types. The sequences were characterized and fed into a graph neural network to obtain corresponding outputs. These trained models were then applied to real and simulated sequence data. Created in BioRender. Ming, F. (2025) https://BioRender.com/x57l607.
(TIF)

S2 Fig Performance of Geno-GNN for diverse types of SARS-CoV-2 fitness. (A) Internal validation for Geno-GNN using ten-fold cross-validation. Each point represents the Spearman’s correlation coefficient between predictions and observations on the testing data. (B-C) External validation for ACE2 binding affinity. The external validation data were collected from the studies of Taylor et al. [47,48] (B) and Moulana et al. [23] (C). (D) Performance of a naive additive model on the same dataset as in (C), serving as a baseline to evaluate the epistatic capture capability. This naive additive model predicts ACE2 affinity by summing the individual effect values of each mutation, where each value represents the experimentally measured change in affinity relative to the wild-type baseline. (E-H) External validation for immune escape using neutralization assay data [3] from diverse immune types: WT vaccine (E), BA.1 + BTI convalescent (F), BA.2 + BTI convalescent (G), and BA.5 + BTI convalescent (H). Each dot represents an Omicron subvariant. The x-axis represents the model’s predicted values, while the y-axis represents the experimental values. The Spearman correlation coefficients and their *p*-values are labeled. The shaded region represents the 95% confidence interval, and the surrounding bar graphs represent the data distribution for the corresponding axis.
(TIF)

S3 Fig Performance of the site-independent escape calculator as a comparative baseline. External validation for the site-independent escape calculator, using the same experimental neutralization assay data [42] as in S2E–H Fig for a direct comparison. Predictions from the calculator were generated with the following key settings: the study was set to ‘Cao et al, 2022, Nature’; the reference variant for neutralization was set to ‘BA.1’; weighting by negative log IC50 and re-weighting of antibodies from non-representative sources were disabled; and the mutation escape strength was set to 2. The validation was performed across diverse immune types: WT vaccine (A), BA.1 + BTI convalescent (B), BA.2 + BTI convalescent (C), and BA.5 + BTI convalescent (D). Each dot represents an Omicron subvariant. The x-axis represents the calculated binding scores, while the y-axis represents the experimental values. The Spearman correlation coefficients and their *p*-values are labeled. The shaded region represents the 95% confidence interval, and the surrounding bar graphs represent the data distribution for the corresponding axis.
(TIF)

S4 Fig Site-level performance of Geno-GNN. (A-F) Comparison of diverse fitness at the RBD site-level between experimental data and predicted results. The blue line represents experimental data; the orange line represents predicted results.
(TIF)

S5 Fig The monthly prevalence proportion of the SARS-CoV-2 macro lineages. The x-axis represents time on a monthly basis from March 2020 to February 2024, and the y-axis represents the proportion of the sampled sequences. The color gradients reflect the main variants for each month.
(TIF)

S6 Fig Comparison of the difference in the magnitude of the change in escape score between the two rapid growth period for WT convalescent and WT vaccine. The Wilcoxon-test results for the difference in the magnitude of change in escape scores between WT convalescent and WT vaccine during the first rapid growth phase (A) and the second rapid growth phase (B) across 100 sampling results. The y-axis represents the fitness values of the sampled sequences. The boxes represent the range between the 25th and 75th percentiles, and the whiskers extend to no further than 1.5 times the inter-quartile range from the largest/smallest value. *p*-values are indicated: *p-value < 0.10; **p-value < 0.05; ***p-value < 0.01; ****p-value < 0.001. NS, not significant.
(TIF)

S7 Fig The monthly changes in fitness values of the sampled sequences. (A, B, C) Temporal changes in immune escape against BA.1 + BTI convalescent (A), BA.2 + BTI convalescent (B), BA.5 + BTI convalescent (C). The x-axis represents monthly time points from March 2020 to February 2024, and the y-axis represents the average fitness values of the sampled sequences. Scores less than 0 were set to 0. The color gradients reflect the predominant variant for each month, defined as the variant with the highest proportion in a given month.
(TIF)

S8 Fig Clustering of SARS-CoV-2 fitness across countries based on Geno-GNN predictions, and its association with administered vaccine types. (A) Heatmap of SARS-CoV-2 fitness across countries before the Omicron wave, with hierarchical clustering represented as a dendrogram on the left. Squares on the left indicate the vaccine types administered in each country. Fitness values are normalized for each fitness category. It is important to note that while this heatmap illustrates an association between fitness clusters and vaccine platforms, our multivariable regression analysis (Main Text and S2 Table) show that this pattern is driven by multiple factors, with vaccine type being one of several statistically significant contributors. (B) Distribution of vaccine types administered across countries. In accordance with a previous study [67], the countries were categorized into four immune backgrounds based on the vaccine platform used: mRNA, adenovirus vector, inactivated, and mixed. The base map layer showing country boundaries was sourced from Natural Earth’s medium-scale (1:50m) cultural vectors (https://www.naturalearthdata.com/downloads/50m-cultural-vectors/). The shapefile data is in the public domain and is freely available for personal and commercial use under the terms of use outlined at: https://www.naturalearthdata.com/about/terms-of-use/.
(TIF)

S9 Fig Temporal change of SARS-CoV-2 fitness grouped by countries with diverse vaccines administered. (A, C, E) Temporal changes in ACE2 binding affinity and immune escape against WT convalescent and WT inactive vaccine across different vaccine administration regions. (B, D, F) The Wilcoxon-test results for the difference in the magnitude of fitness change between countries where different vaccines were administered. The y-axis represents the average fitness values of the sampled sequences. The color gradients represent the countries with different types of vaccines. *p*-values are indicated: *p-value < 0.10; **p-value < 0.05; ***p-value < 0.01; ****p-value < 0.001. NS, not significant.
(TIF)

S10 Fig Fitness landscape of fixed mutations. The distribution of ACE2 binding affinities is grouped by the number of mutations and the number of immune types escaped. The color gradient reflects the number of escaped immune types. The black dashed line represents the ACE2 affinity of the wildtype. The boxes represent the range between the 25th and 75th percentiles, with whiskers extending to 1.5 times the interquartile range. “Fixed mutations” refers to the set of mutations shared by all studied variants. To evaluate the impact of each individual fixed mutation on fitness, we conducted a separate virtual mutation scanning analysis, treating each fixed mutation separately. This approach contrasts with the evolutionary trajectory analysis, where fixed mutations were considered co-mutated during virtual mutation scanning.
(TIF)

S1 Table Characteristics of data collected. (XLSX)

S2 Table Multiple regression analysis of factors associated with viral fitness levels. (XLSX)

S3 Table Fitness change rates across regions for 8 time periods. Since the Alpha dominance period lasted only one month, and the Delta-to-Omicron transition was only two months, a reliable regression cannot be performed, resulting in null values in the table.
(XLSX)

S4 Table The *p*-values from the slope difference test for comparing piecewise regression coefficients across regions over 8 time periods. (XLSX)

S5 Table Multiple regression analysis of factors associated with the rate of fitness change during the Others-to-Alpha transition period. (XLSX)

S6 Table Multiple regression analysis of factors associated with the rate of fitness change during the Alpha-to-Delta transition period. (XLSX)

S7 Table RBD mutations of Omicron subvariants. (XLSX)
