# Supplementary material for: Characterization of the heterogeneity in SARS-CoV-2 fitness dynamics via graph representation learning
Source: PLoS Comput Biol. 2026 Jan 12;22(1):e1013582. doi: 10.1371/journal.pcbi.1013582 (PMC12810920; doi:10.1371/journal.pcbi.1013582)
Supplement: S1 Text — (DOCX) [file pcbi.1013582.s001.docx]

**S1 Text. The framework of Geno-GNN and its performance for six types of virus fitness**

Our study began with the compilation of deep mutational scanning (DMS) data for antibodies obtained from human sera with diverse immunity types[1–3] (Table S1 and Methods), including WT convalescent (infected with wild-type SARS-CoV-2), WT inactive vaccine (received three doses of CoronaVac), BA.1+BTI convalescent (infected with BA.1 after three doses of CoronaVac), BA.2+BTI convalescent (infected with BA.2 after three doses of CoronaVac), and BA.5+BTI convalescent (infected with BA.5 after three doses of CoronaVac). For each RBD variant, escape scores across all available antibodies from individuals with matching immunity type were aggregated. We assumed that averaging escape score across antibodies with the same source would effectively represent the characteristics of actual human sera, a hypothesis used in previous study[4]. Additionally, DMS data for ACE2 binding affinity were also collected[5,6], enabling the analysis of six types of SARS-CoV-2 fitness in our study.

We devised a deep learning model named Geno-GNN for predicting various types of fitness, using amino acid sequence of the RBD as input (S1 Fig, Methods). We characterized each amino acid employing 8 principal components derived from 50 physicochemical variables, encompassing hydrophobic, steric, and electronic properties[7]. These amino acid eigenvectors were sequentially connected to form an undirected graph, serving as the input feature module for the GNN model. The information was passed in the input graph, allowing the model to capture the intricate effects of high dimensional combinatorial mutations on the SARS-CoV-2 fitness.

To assess the performance of Geno-GNN, 10-fold cross-validation was employed (S2A Fig). For ACE2 binding affinity, the Spearman correlation between the predictions and the observations on the testing dataset was 0.952 (95%CI: 0.949-0.955). Regarding immune escape, the correlation ranged from 0.668 (95%CI: 0.619-0.716) in BA.5+BTI convalescent to 0.755 (95%CI: 0.734-0.775) in BA.2+BTI convalescent (S2A Fig). The models exhibited lower performance for immune escape, potentially due to the limited sample size relative to ACE2 binding affinity data (S1 Table). Furthermore, Geno-GNN demonstrated robust performance at the RBD site level, with a Spearman correlation of 0.99 for ACE2 binding affinity (S4A Fig). For immune escape, the site-level correlations ranged from 0.92 for WT inactive vaccine to 0.94 for BA.2+BTI convalescent (S4B-F Fig).

To further validate ACE2 affinity, we incorporated external DMS experiments from previous studies of Taylor et al.[8,9] and Moulana et al.[10], and Geno-GNN achieved correlations of 0.88 and 0.86, respectively (S2B-C Fig). For immune escape, strong correlations between predicted escape scores and external neutralization titers across diverse immune backgrounds were observed (*R*=-0.75, *p* < 0.001 for WT inactive vaccine; *R*=-0.86, *p* < 0.001 for BA.1+BTI convalescent; *R*=-0.82, *p* < 0.001 for BA.2+BTI convalescent; *R*=-0.64, *p* < 0.001 for BA.5+BTI convalescent, S2E-H Fig). These results underscored the efficacy of Geno-GNN in predicting various aspects of SARS-CoV-2 fitness.

**References**

1. Cao Y, Yisimayi A, Jian F, Song W, Xiao T, Wang L, et al. BA.2.12.1, BA.4 and BA.5 escape antibodies elicited by Omicron infection. Nature. 2022;608: 593–602. doi:10.1038/s41586-022-04980-y

2. Cao Y, Wang J, Jian F, Xiao T, Song W, Yisimayi A, et al. Omicron escapes the majority of existing SARS-CoV-2 neutralizing antibodies. Nature. 2022;602: 657–663. doi:10.1038/s41586-021-04385-3

3. Cao Y, Jian F, Wang J, Yu Y, Song W, Yisimayi A, et al. Imprinted SARS-CoV-2 humoral immunity induces convergent Omicron RBD evolution. Nature. 2023;614: 521–529. doi:10.1038/s41586-022-05644-7

4. Greaney AJ, Starr TN, Bloom JD. An antibody-escape estimator for mutations to the SARS-CoV-2 receptor-binding domain. Virus Evol. 2022;8: veac021. doi:10.1093/ve/veac021

5. Starr TN, Greaney AJ, Hannon WW, Loes AN, Hauser K, Dillen JR, et al. Shifting mutational constraints in the SARS-CoV-2 receptor-binding domain during viral evolution. Science. 2022;377: 420–424. doi:10.1126/science.abo7896

6. Starr TN, Greaney AJ, Stewart CM, Walls AC, Hannon WW, Veesler D, et al. Deep mutational scans for ACE2 binding, RBD expression, and antibody escape in the SARS-CoV-2 Omicron BA.1 and BA.2 receptor-binding domains. Mok CKP, editor. PLoS Pathog. 2022;18: e1010951. doi:10.1371/journal.ppat.1010951

7. Mei H, Liao ZH, Zhou Y, Li SZ. A new set of amino acid descriptors and its application in peptide QSARs. Biopolymers. 2005;80: 775–786. doi:10.1002/bip.20296

8. Taylor AL, Starr TN. Deep mutational scans of XBB.1.5 and BQ.1.1 reveal ongoing epistatic drift during SARS-CoV-2 evolution. Evolutionary Biology; 2023 Sept. doi:10.1101/2023.09.11.557279

9. Taylor AL, Starr TN. Deep mutational scanning of SARS-CoV-2 Omicron BA.2.86 and epistatic emergence of the KP.3 variant. Virus Evol. 2024;10: veae067. doi:10.1093/ve/veae067

10. Moulana A, Dupic T, Phillips AM, Chang J, Nieves S, Roffler AA, et al. Compensatory epistasis maintains ACE2 affinity in SARS-CoV-2 Omicron BA.1. Nat Commun. 2022;13: 7011. doi:10.1038/s41467-022-34506-z
