# Supplementary figures and images for: Characterization of the heterogeneity in SARS-CoV-2 fitness dynamics via graph representation learning
Source: PLoS Comput Biol. 2026 Jan 12;22(1):e1013582. doi: 10.1371/journal.pcbi.1013582 (PMC12810920; doi:10.1371/journal.pcbi.1013582)

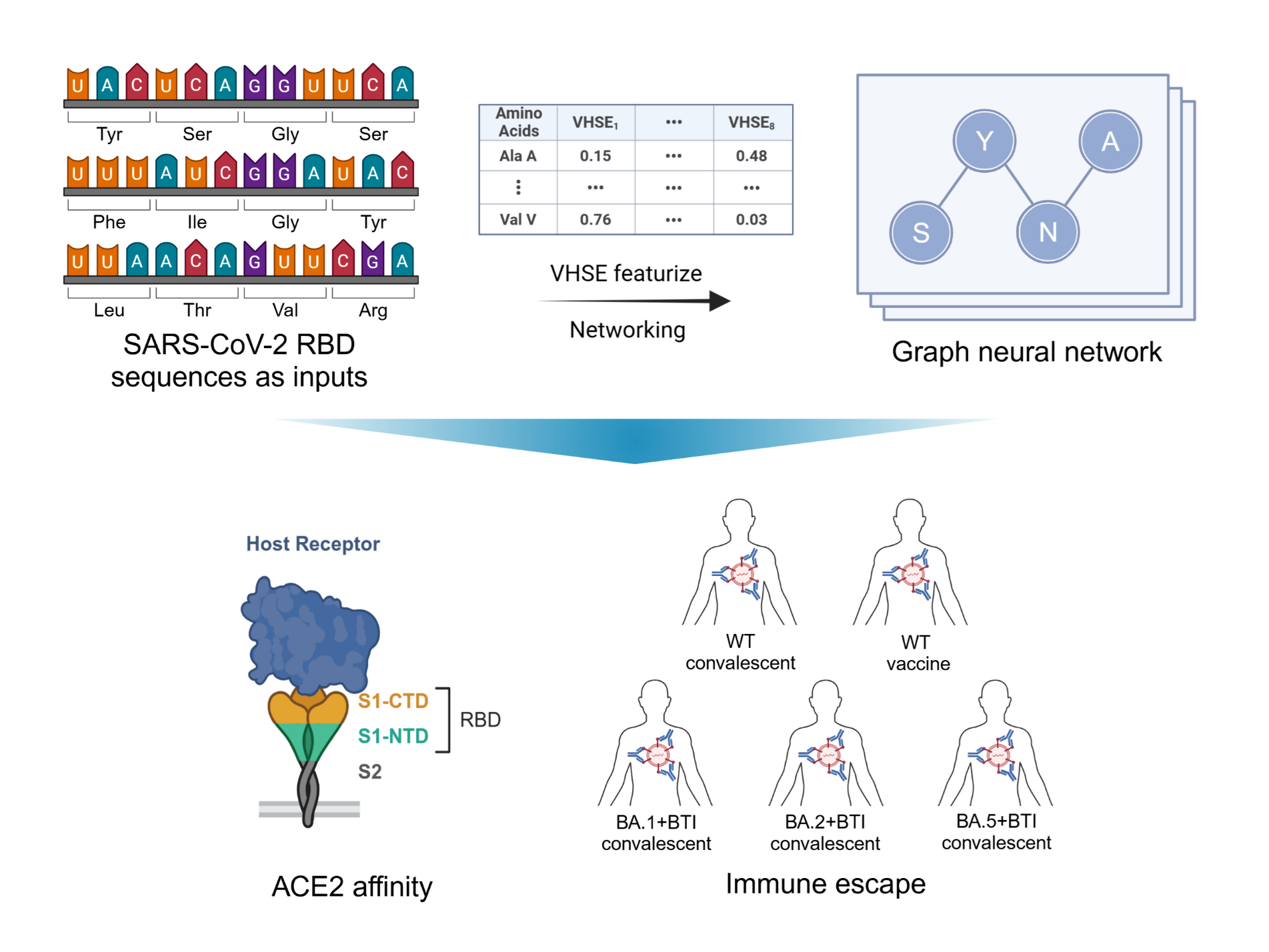

Supplement: S1 Fig — We collected RBD variant sequences from deep mutational scanning data along with their corresponding ACE2 affinity and their ability to escape various immune types. The sequences were characterized and fed into a graph neural network to obtain corresponding outputs. These trained models were then applied to real and simulated sequence data. Created in BioRender. Ming, F. (2025) https://BioRender.com/x57l607. (TIF) [file pcbi.1013582.s002.tif]

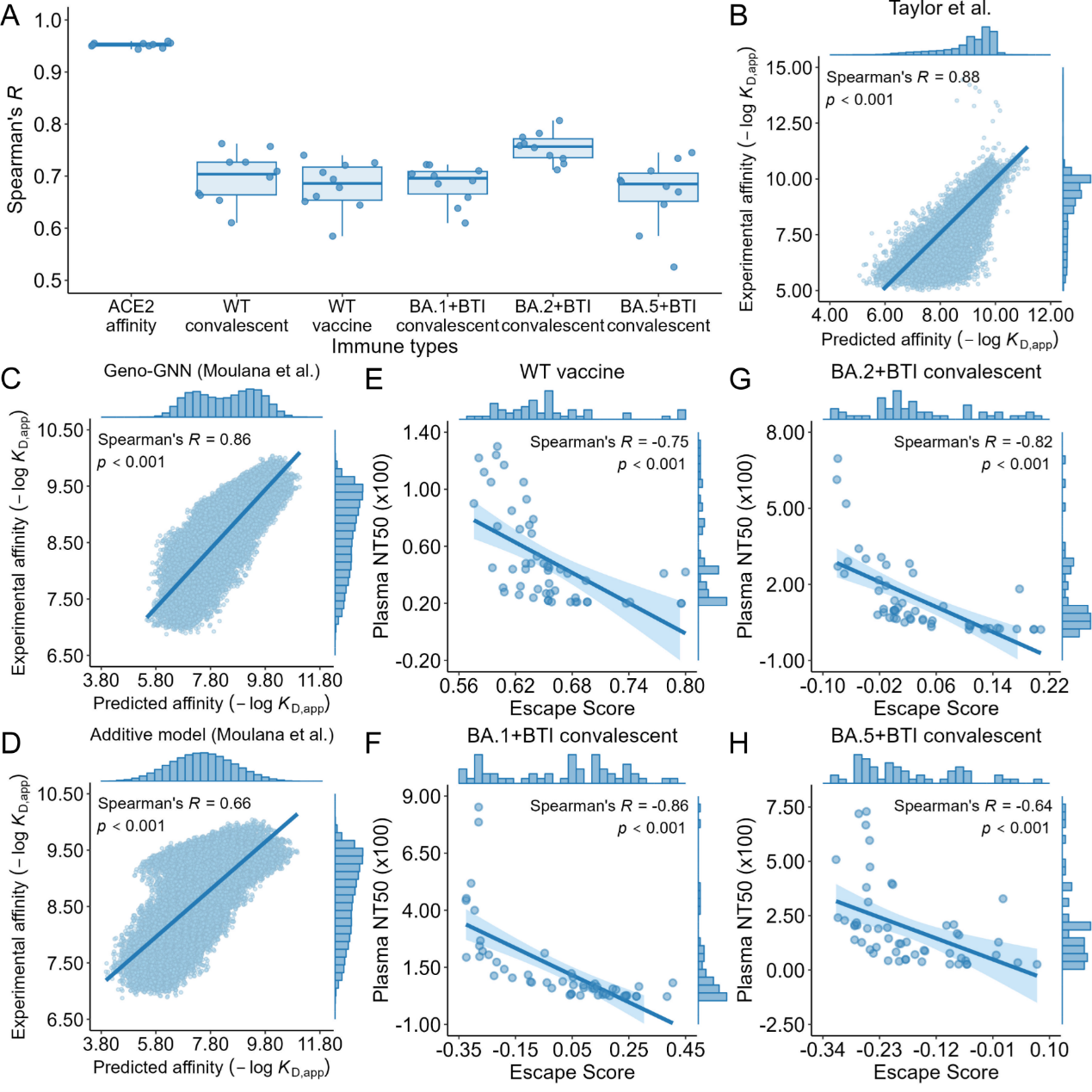

Supplement: S2 Fig — (A) Internal validation for Geno-GNN using ten-fold cross-validation. Each point represents the Spearman’s correlation coefficient between predictions and observations on the testing data. (B-C) External validation for ACE2 binding affinity. The external validation data were collected from the studies of Taylor et al. [47,48] (B) and Moulana et al. [23] (C). (D) Performance of a naive additive model on the same dataset as in (C), serving as a baseline to evaluate the epistatic capture capability. This naive additive model predicts ACE2 affinity by summing the individual effect values of each mutation, where each value represents the experimentally measured change in affinity relative to the wild-type baseline. (E-H) External validation for immune escape using neutralization assay data [3] from diverse immune types: WT vaccine (E), BA.1 + BTI convalescent (F), BA.2 + BTI convalescent (G), and BA.5 + BTI convalescent (H). Each dot represents an Omicron subvariant. The x-axis represents the model’s predicted values, while the y-axis represents the experimental values. The Spearman correlation coefficients and their p-values are labeled. The shaded region represents the 95% confidence interval, and the surrounding bar graphs represent the data distribution for the corresponding axis. (TIF) [file pcbi.1013582.s003.tif]

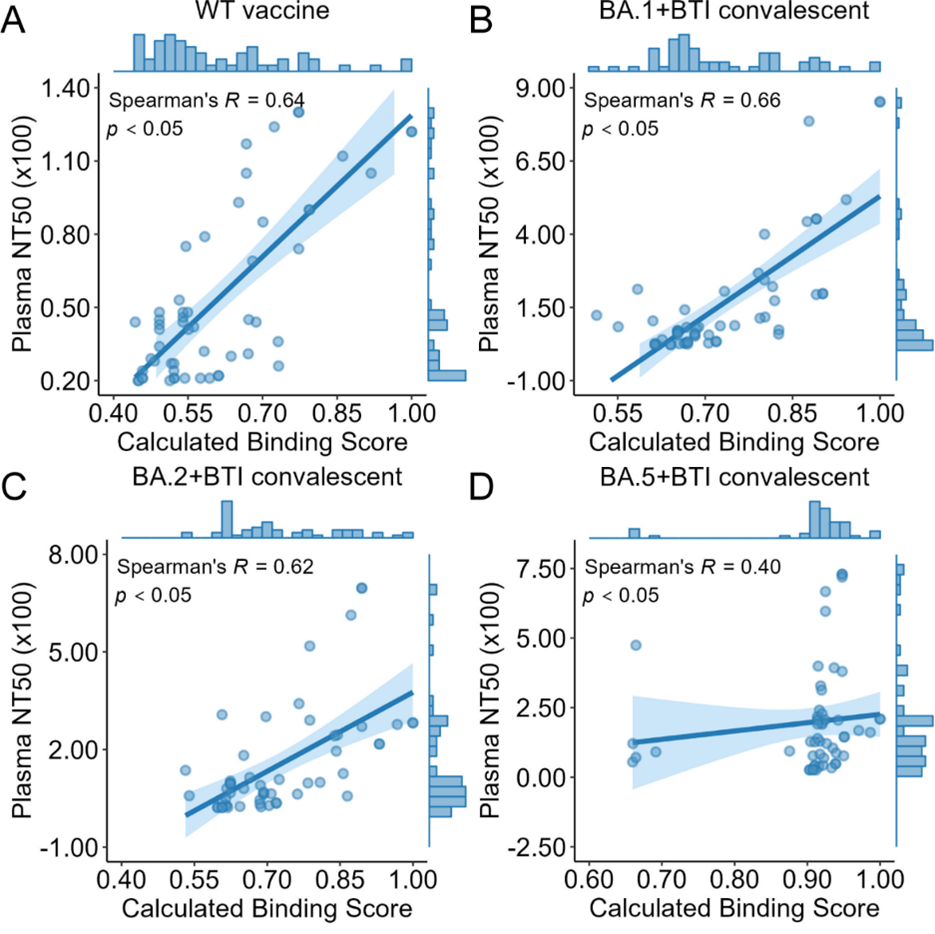

Supplement: S3 Fig — External validation for the site-independent escape calculator, using the same experimental neutralization assay data [42] as in S2E–H Fig for a direct comparison. Predictions from the calculator were generated with the following key settings: the study was set to ‘Cao et al, 2022, Nature’; the reference variant for neutralization was set to ‘BA.1’; weighting by negative log IC50 and re-weighting of antibodies from non-representative sources were disabled; and the mutation escape strength was set to 2. The validation was performed across diverse immune types: WT vaccine (A), BA.1 + BTI convalescent (B), BA.2 + BTI convalescent (C), and BA.5 + BTI convalescent (D). Each dot represents an Omicron subvariant. The x-axis represents the calculated binding scores, while the y-axis represents the experimental values. The Spearman correlation coefficients and their p-values are labeled. The shaded region represents the 95% confidence interval, and the surrounding bar graphs represent the data distribution for the corresponding axis. (TIF) [file pcbi.1013582.s004.tif]

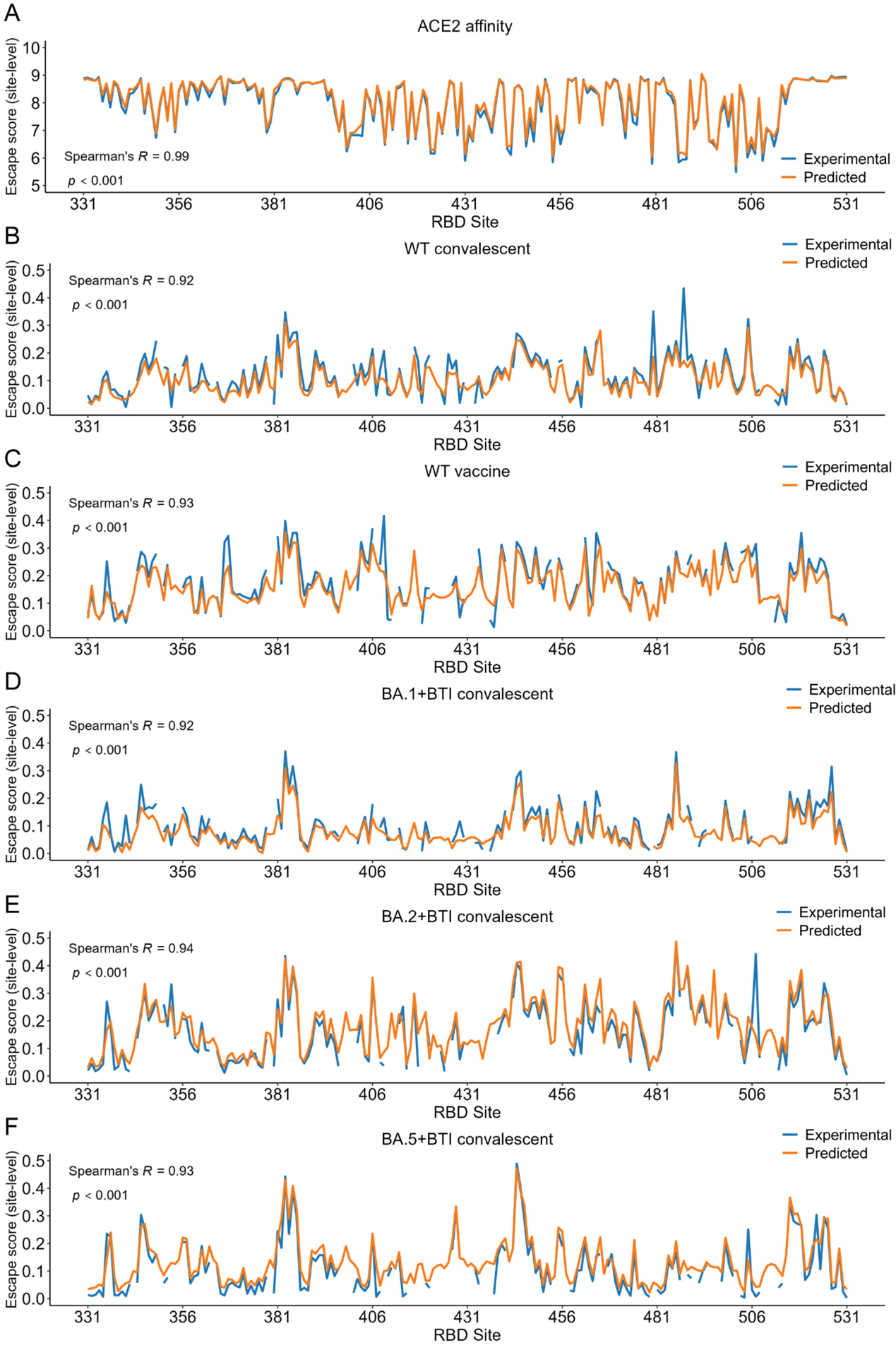

Supplement: S4 Fig — (A-F) Comparison of diverse fitness at the RBD site-level between experimental data and predicted results. The blue line represents experimental data; the orange line represents predicted results. (TIF) [file pcbi.1013582.s005.tif]

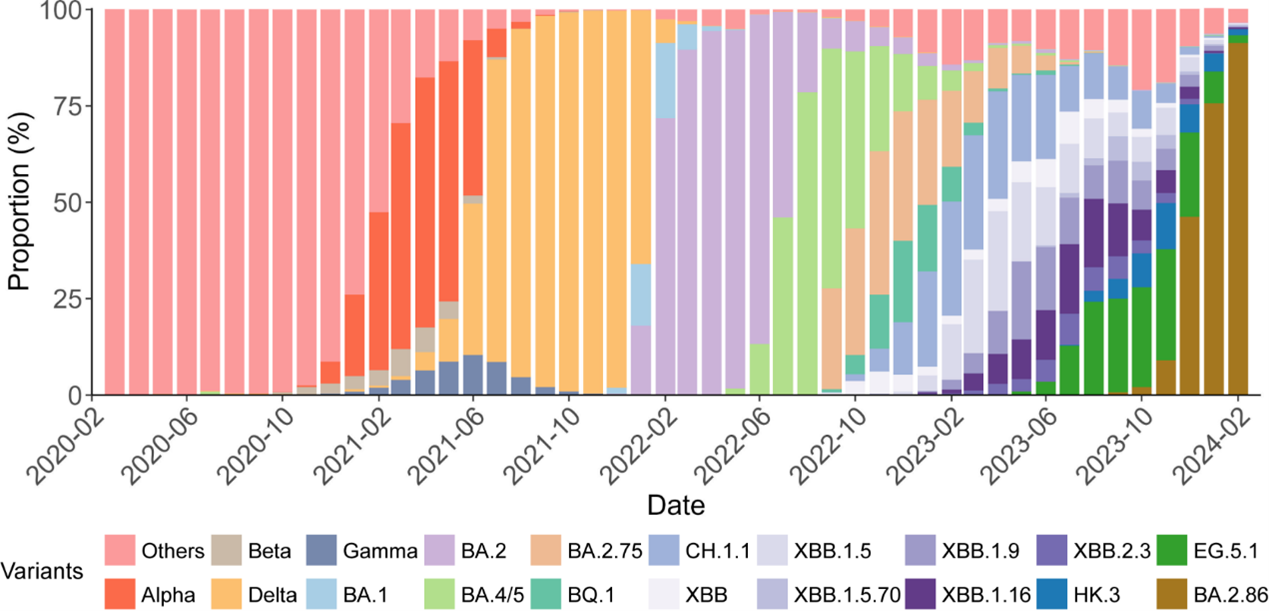

Supplement: S5 Fig — The x-axis represents time on a monthly basis from March 2020 to February 2024, and the y-axis represents the proportion of the sampled sequences. The color gradients reflect the main variants for each month. (TIF) [file pcbi.1013582.s006.tif]

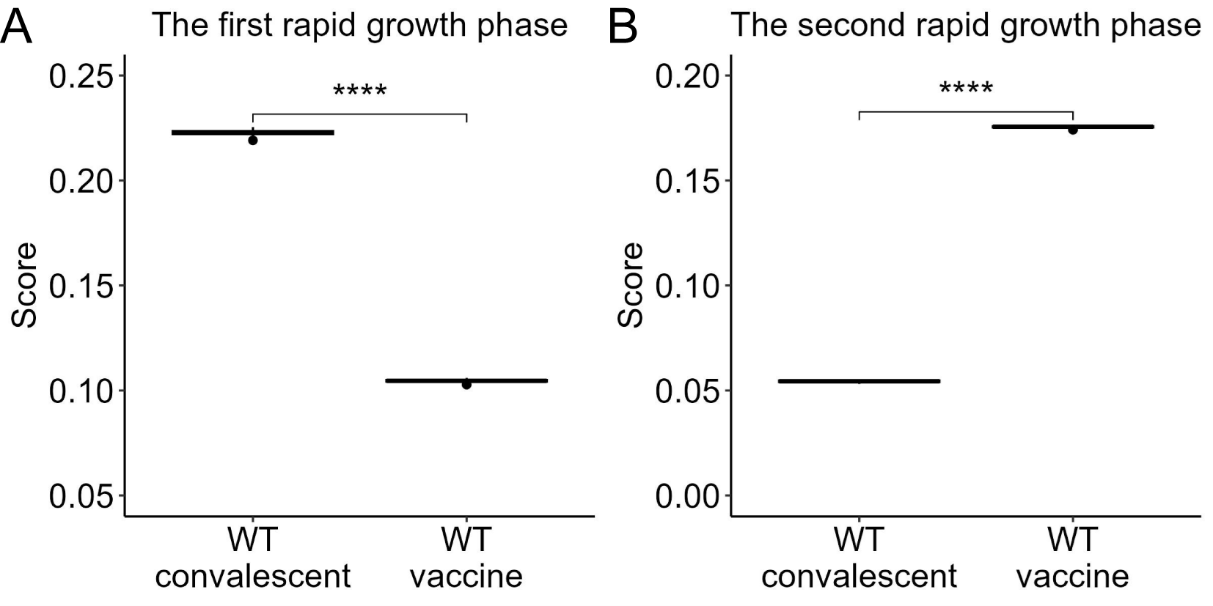

Supplement: S6 Fig — The Wilcoxon-test results for the difference in the magnitude of change in escape scores between WT convalescent and WT vaccine during the first rapid growth phase (A) and the second rapid growth phase (B) across 100 sampling results. The y-axis represents the fitness values of the sampled sequences. The boxes represent the range between the 25th and 75th percentiles, and the whiskers extend to no further than 1.5 times the inter-quartile range from the largest/smallest value. p-values are indicated: *p-value < 0.10; **p-value < 0.05; ***p-value < 0.01; ****p-value < 0.001. NS, not significant. (TIF) [file pcbi.1013582.s007.tif]

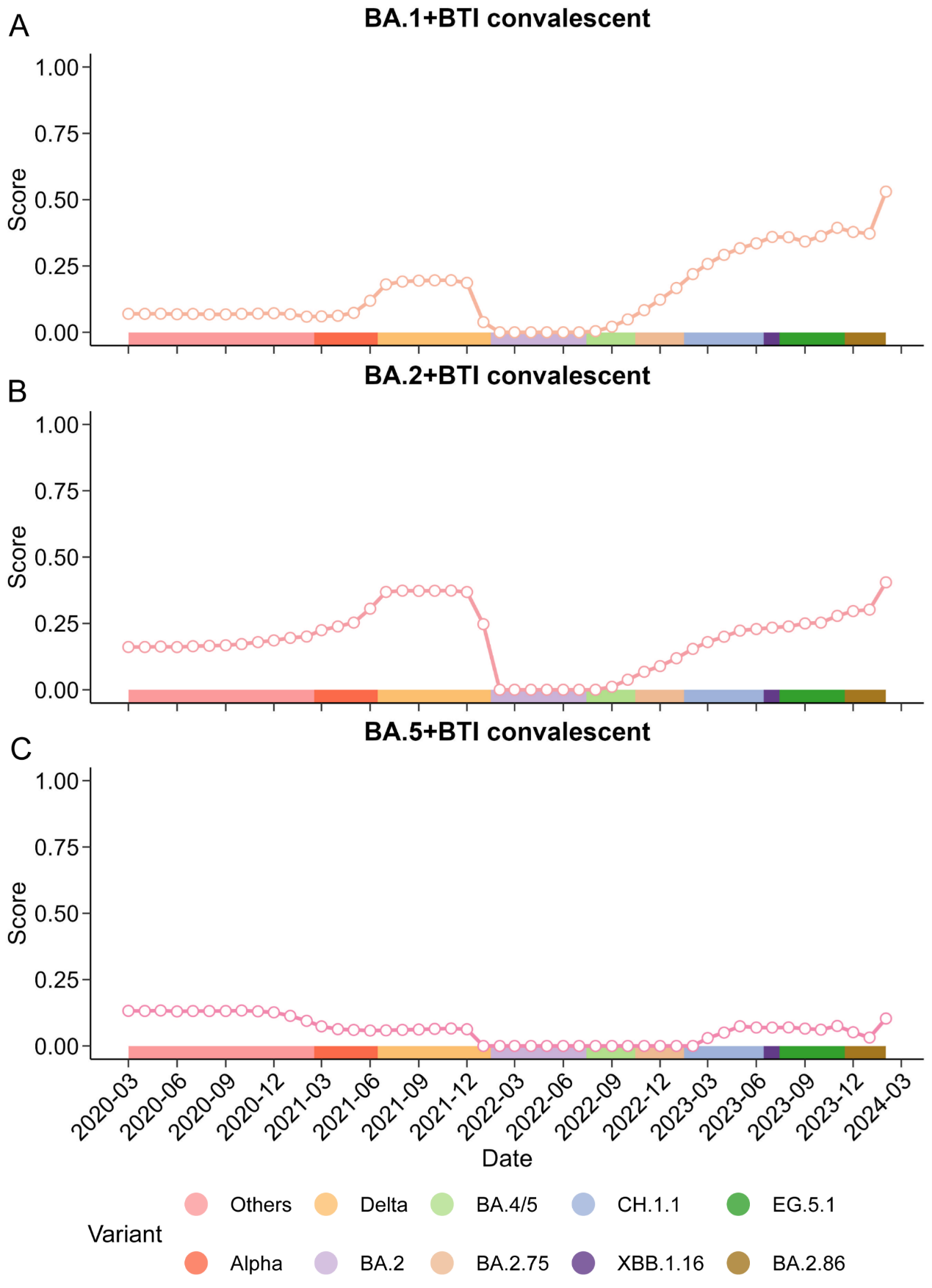

Supplement: S7 Fig — (A, B, C) Temporal changes in immune escape against BA.1 + BTI convalescent (A), BA.2 + BTI convalescent (B), BA.5 + BTI convalescent (C). The x-axis represents monthly time points from March 2020 to February 2024, and the y-axis represents the average fitness values of the sampled sequences. Scores less than 0 were set to 0. The color gradients reflect the predominant variant for each month, defined as the variant with the highest proportion in a given month. (TIF) [file pcbi.1013582.s008.tif]

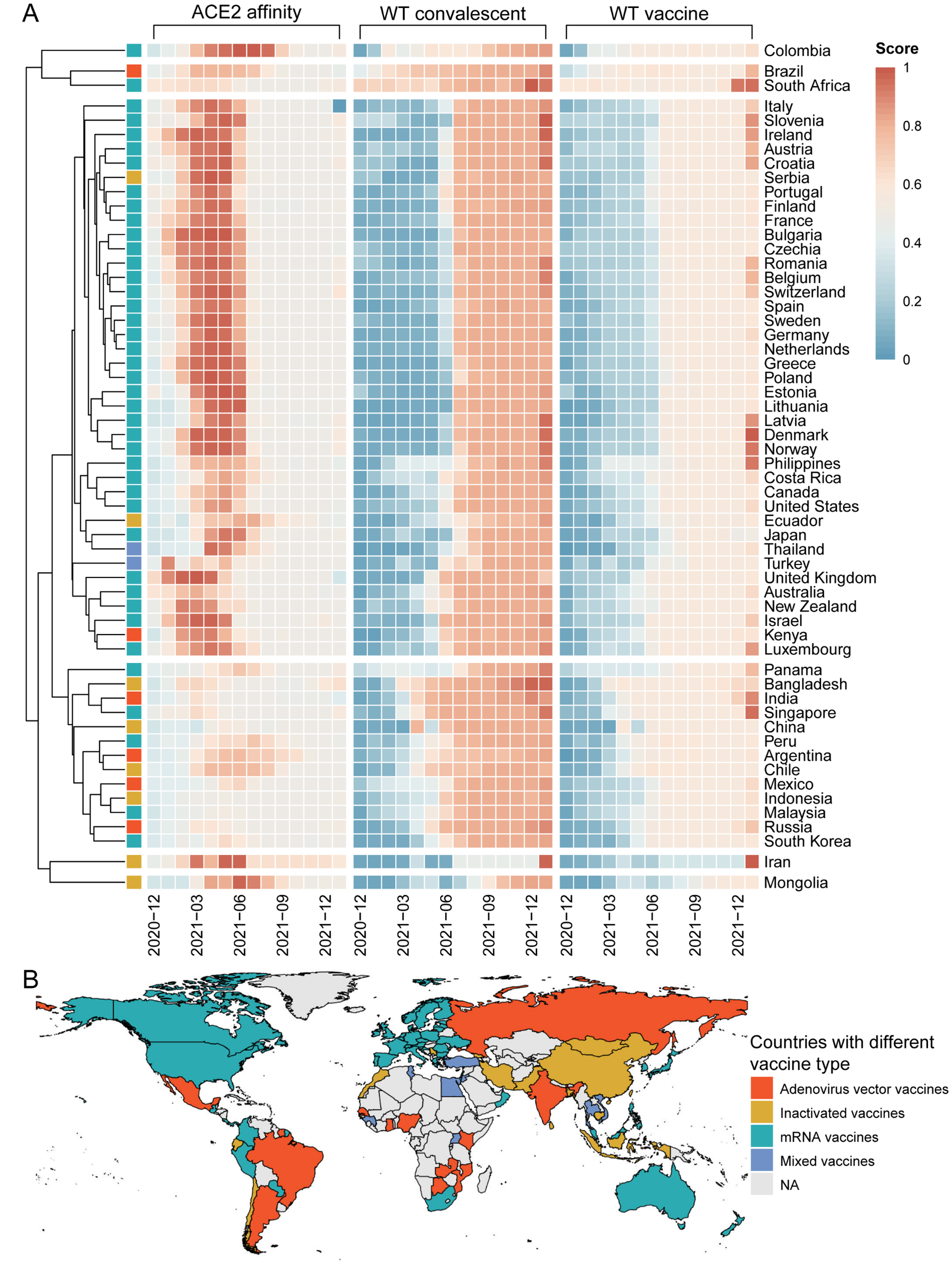

Supplement: S8 Fig — (A) Heatmap of SARS-CoV-2 fitness across countries before the Omicron wave, with hierarchical clustering represented as a dendrogram on the left. Squares on the left indicate the vaccine types administered in each country. Fitness values are normalized for each fitness category. It is important to note that while this heatmap illustrates an association between fitness clusters and vaccine platforms, our multivariable regression analysis (Main Text and S2 Table) show that this pattern is driven by multiple factors, with vaccine type being one of several statistically significant contributors. (B) Distribution of vaccine types administered across countries. In accordance with a previous study [67], the countries were categorized into four immune backgrounds based on the vaccine platform used: mRNA, adenovirus vector, inactivated, and mixed. The base map layer showing country boundaries was sourced from Natural Earth’s medium-scale (1:50m) cultural vectors (https://www.naturalearthdata.com/downloads/50m-cultural-vectors/). The shapefile data is in the public domain and is freely available for personal and commercial use under the terms of use outlined at: https://www.naturalearthdata.com/about/terms-of-use/. (TIF) [file pcbi.1013582.s009.tif]

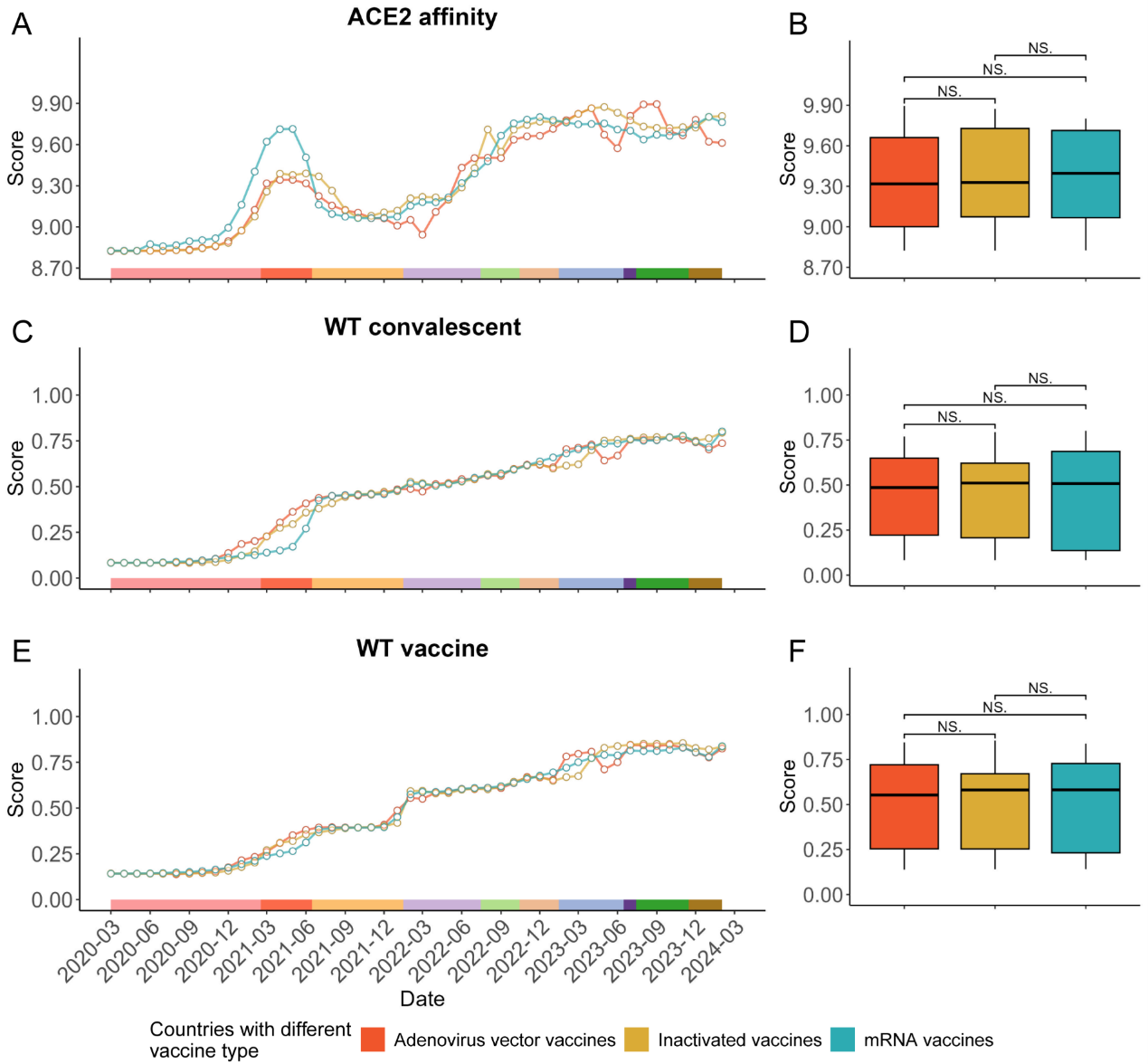

Supplement: S9 Fig — (A, C, E) Temporal changes in ACE2 binding affinity and immune escape against WT convalescent and WT inactive vaccine across different vaccine administration regions. (B, D, F) The Wilcoxon-test results for the difference in the magnitude of fitness change between countries where different vaccines were administered. The y-axis represents the average fitness values of the sampled sequences. The color gradients represent the countries with different types of vaccines. p-values are indicated: *p-value < 0.10; **p-value < 0.05; ***p-value < 0.01; ****p-value < 0.001. NS, not significant. (TIF) [file pcbi.1013582.s010.tif]

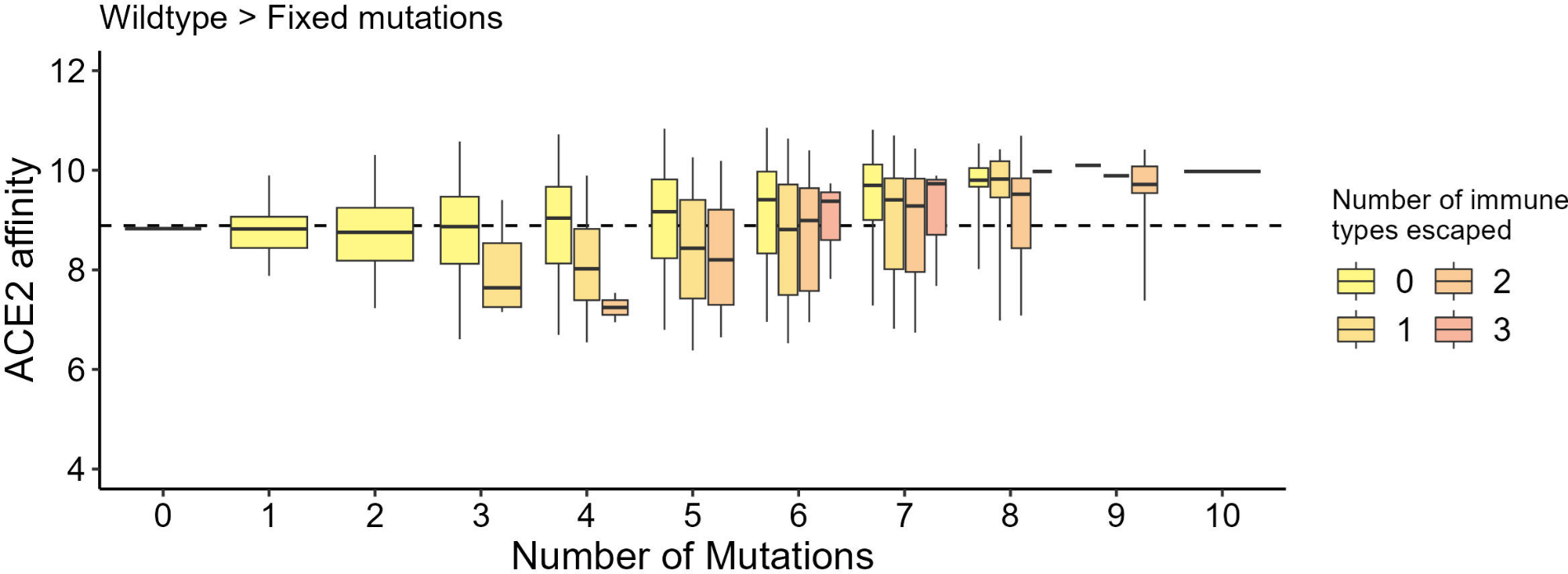

Supplement: S10 Fig — The distribution of ACE2 binding affinities is grouped by the number of mutations and the number of immune types escaped. The color gradient reflects the number of escaped immune types. The black dashed line represents the ACE2 affinity of the wildtype. The boxes represent the range between the 25th and 75th percentiles, with whiskers extending to 1.5 times the interquartile range. “Fixed mutations” refers to the set of mutations shared by all studied variants. To evaluate the impact of each individual fixed mutation on fitness, we conducted a separate virtual mutation scanning analysis, treating each fixed mutation separately. This approach contrasts with the evolutionary trajectory analysis, where fixed mutations were considered co-mutated during virtual mutation scanning. (TIF) [file pcbi.1013582.s011.tif]
